# Microarray Gene Expression Analysis to Evaluate Cell Type Specific Expression of Targets Relevant for Immunotherapy of Hematological Malignancies

**DOI:** 10.1371/journal.pone.0155165

**Published:** 2016-05-12

**Authors:** M. J. Pont, M. W. Honders, A. N. Kremer, C. van Kooten, C. Out, P. S. Hiemstra, H. C. de Boer, M. J. Jager, E. Schmelzer, R. G. Vries, A. S. Al Hinai, W. G. Kroes, R. Monajemi, J. J. Goeman, S. Böhringer, W. A. F. Marijt, J. H. F. Falkenburg, M. Griffioen

**Affiliations:** 1 Department of Hematology, Leiden University Medical Center, Leiden, the Netherlands; 2 Department of Internal Medicine 5, Hematology and Oncology, University Hospital Erlangen, Erlangen, Germany; 3 Department of Nephrology, Leiden University Medical Center, Leiden, the Netherlands; 4 Department of Dermatology, Leiden University Medical Center, Leiden, the Netherlands; 5 Department of Pulmonology, Leiden University Medical Center, Leiden, the Netherlands; 6 Department of Nephrology and the Einthoven Laboratory for Experimental Vascular Medicine, Leiden University Medical Center, Leiden, the Netherlands; 7 Department of Ophthalmology, Leiden University Medical Center, Leiden, The Netherlands; 8 McGowan Institute for Regenerative Medicine, University of Pittsburgh, Pittsburgh, Pennsylvania, United States of America; 9 Hubrecht Institute for Developmental Biology and Stem Cell Research and University Medical Centre Utrecht, Utrecht, the Netherlands; 10 Department of Hematology, Erasmus University Medical Center Cancer Institute, Rotterdam, the Netherlands; 11 Department of Clinical Genetics, Leiden University Medical Center, Leiden, The Netherlands; 12 Department of Medical Statistics and Bioinformatics, Leiden University Medical Center, Leiden, The Netherlands; 13 Radboud Institute for Molecular Life Science, Radboud University Medical Center, Nijmegen, The Netherlands; INRS, CANADA

## Abstract

Cellular immunotherapy has proven to be effective in the treatment of hematological cancers by donor lymphocyte infusion after allogeneic hematopoietic stem cell transplantation and more recently by targeted therapy with chimeric antigen or T-cell receptor-engineered T cells. However, dependent on the tissue distribution of the antigens that are targeted, anti-tumor responses can be accompanied by undesired side effects. Therefore, detailed tissue distribution analysis is essential to estimate potential efficacy and toxicity of candidate targets for immunotherapy of hematological malignancies. We performed microarray gene expression analysis of hematological malignancies of different origins, healthy hematopoietic cells and various non-hematopoietic cell types from organs that are often targeted in detrimental immune responses after allogeneic stem cell transplantation leading to graft-versus-host disease. Non-hematopoietic cells were also cultured in the presence of IFN-γ to analyze gene expression under inflammatory circumstances. Gene expression was investigated by Illumina HT12.0 microarrays and quality control analysis was performed to confirm the cell-type origin and exclude contamination of non-hematopoietic cell samples with peripheral blood cells. Microarray data were validated by quantitative RT-PCR showing strong correlations between both platforms. Detailed gene expression profiles were generated for various minor histocompatibility antigens and B-cell surface antigens to illustrate the value of the microarray dataset to estimate efficacy and toxicity of candidate targets for immunotherapy. In conclusion, our microarray database provides a relevant platform to analyze and select candidate antigens with hematopoietic (lineage)-restricted expression as potential targets for immunotherapy of hematological cancers.

## Introduction

Cellular immunotherapy of hematological cancers has proven very effective. After allogeneic hematopoietic stem cell transplantation (alloSCT), anti-tumor immunity is mediated by donor T cells recognizing the malignant cells of the patient [1]. Another effective approach is targeted therapy by chimeric antigen receptor (CAR) or T-cell receptor (TCR) gene transfer. CAR T-cell therapy specific for CD19 has successfully been used to treat patients with B-cell malignancies [2]. In addition to strong anti-tumor immunity, immunotherapy can cause life-threatening toxicity, i.e. liver or neurological damage as reported after CAR or TCR gene therapy [3, 4] or graft-versus-host disease (GvHD) after alloSCT [5], due to on-target recognition of healthy organs by the adoptively transferred T cells. Both the efficacy and potential toxicity of immunotherapy is strongly dependent on the tissue distribution of the antigens that are targeted. Thus, gene expression profiles of candidate targets for immunotherapy of hematological cancers need to be carefully examined.

Immunotherapy can be directed against extracellular or intracellular antigens. Specific antibodies or CARs can recognize extracellular antigens that are expressed on the cell surface of malignant cells. These antigens need to be selectively expressed on the tumor or on the lineage from which the tumor originates to limit the risk of toxicity [2, 6]. Intracellular antigens can be targeted by specific TCRs when peptides from these proteins are presented by HLA on the cell surface. As such, the repertoire of candidate antigens that can be targeted by TCR-based immunotherapy extends beyond extracellular antigens, but the necessity for tumor- or lineage-restricted expression remains. In the setting of alloSCT, polymorphic antigens with hematopoietic-restricted expression are relevant targets for immunotherapy, since donor T cells recognizing these antigens eliminate the malignant cells of the patient, while sparing healthy hematopoietic cells of donor origin. Polymorphic peptides that are targeted by donor T cells after HLA-matched alloSCT, so-called minor histocompatibility antigens, can be efficiently discovered by whole genome association scanning and minor histocompatibility antigens with hematopoiesis-restricted expression are selected as targets with potential therapeutic relevance [7–11]. Ideally, the tissue distribution of minor histocompatibility antigens is analyzed by measuring T-cell recognition of a large variety of (malignant) hematopoietic and non-hematopoietic cell types cultured from tissues that are targeted in GvHD. However, non-hematopoietic cells are often difficult to culture and not available in quantities that allow in depth T-cell analysis. Therefore, as an alternative, the tissue distribution can be estimated by determining gene expression.

Whole transcriptome analysis can be performed by microarray gene expression or RNA-sequence analysis. Microarray data have become increasingly available over the years in platforms such as Gene Expression Omnibus [12, 13]. However, integration of datasets is challenging due to differences in sample handling and technologies. Various integrated and normalized datasets are offered now and allow online analysis of tissue expression. Oncomine is a large platform providing cancer microarray data [14], while BioGPS, among others, allows easy access to Gene Atlas datasets [15]. GeneSapiens [16] provides a bioinformatic analysis of ~10,000 samples including normal human tissues, different cancer types and cell lines. Many samples in these databases represent whole tissues, which are composed of a mix of non-hematopoietic cell types that are often contaminated with peripheral blood cells. Gene expression profiles from these samples are heterogeneous in nature and do not allow accurate identification of hematopoiesis (lineage)-restricted genes. The value of existing datasets with whole tissue samples for estimating the therapeutic relevance and potential toxicity of candidate targets for immunotherapy of hematological malignancies thus remains limited.

AlloSCT and CAR/TCR gene transfer are treatments that can induce inflammation in patients in particular upon development of an effective anti-tumor response [17–19]. Therefore, to estimate potential toxicity, expression of the antigen targeted by immunotherapy needs to be investigated under inflammatory conditions. However, whole tissues or non-hematopoietic cells are generally collected for transcriptome analysis under non-inflammatory conditions and on line databases are therefore of limited use to estimate potential toxicity of immunotherapeutic targets.

In this study, we performed microarray gene expression analysis on hematological malignancies of different origins isolated from bone marrow or peripheral blood based on expression of specific surface markers. We also collected healthy hematopoietic cells and non-hematopoietic cells from organs that are often targeted in GvHD. Non-hematopoietic cells were cultured from tissue specimen or biopsies and various non-hematopoietic cell types were cultured in the presence of IFN-γ to mimic inflammation. Gene expression was investigated by Illumina HT12.0 microarray and quality control analysis confirmed the cell-type origin of the samples and excluded contamination of non-hematopoietic cell samples with peripheral blood cells. We validated gene expression as measured by microarray gene expression analysis by quantitative RT-PCR and investigated gene expression in non-hematopoietic cells under conditions of inflammation. Finally, we illustrated the value of our dataset to estimate efficacy and toxicity of potential targets for immunotherapy of hematological malignancies.

## Materials and Methods

### Collection of human samples

Malignant and normal hematopoietic cells were isolated from peripheral blood or bone marrow samples and non-hematopoietic cell types were obtained from tissue biopsies or surgically removed specimen obtained from patients or healthy individuals after approval by the Medical Research Ethics Committee and Institutional Review Board of the Leiden University Medical Center (LUMC) and informed consent according to the Declaration of Helsinki. The majority of samples were collected after 2002 and written informed consent was obtained for these samples. Only for leukemic samples collected before 2002, oral informed consent was documented in the patient files by the patient’s physician as the only procedure approved by the ethical board.

### Isolation and culture of healthy hematopoietic cells

Detailed isolation and culture methods are described in S1 Methods. Briefly, bone marrow and peripheral blood mononuclear cells (BMMC and PBMC) were isolated by Ficoll-Isopaque separation and cryopreserved. B cells, T cells, monocytes and hematopoietic stem cells were purified by fluorescence-activated cell sorting. Immature DC (imDC) were generated from isolated monocytes and further differentiated into mature DC (matDC). Isolated monocytes were also used to generate M1 and M2 macrophages (MФ). EBV-transformed B-cell lines (EBV-LCL) and PHA-stimulated T-cell lines (PHA-T) were generated from PBMC as previously described [20, 21].

### Isolation and characterization of malignant hematopoietic cells

Detailed isolation methods are described in S1 Methods. In short, flow cytometric analyses were performed on malignant cells, followed by cell sorting based on surface expression of specific markers. Malignant hematopoietic cells included are acute B-lymphoblastic leukemia (ALL) cells, chronic lymphocytic leukemia (CLL) cells, CD34-positive chronic myeloid leukemia (CML) cells, multiple myeloma (MM) cells and acute myeloid leukemia (AML) cells. AML samples were sorted as single cell populations using antibodies for CD33 or as two separate cell populations expressing CD33 in the absence or presence of CD14 to distinguish cells differentiated into the monocytic pathway (CD14pos) from more immature cells (CD14neg). To classify the different malignant samples according to WHO 2008 standards [22], FISH and karyotyping was performed on freshly isolated or cryopreserved cells. For AML samples, additional analyses were performed to determine *NPM1* mutations and *FLT3* gene internal tandem duplication (*FLT3*-ITD) on genomic DNA and *CEPBA* mutation [23] and *EVI1* overexpression [24, 25] on cDNA.

### Culture of malignant human cell lines

The human erythroleukemia cell line K562, T-B lymphoblastoid cell line T2, acute myeloid leukemia cell lines AML-193 and THP-1, Burkitt's lymphoma cell line Daudi, acute T cell leukemia cell line Jurkat and the human cervix carcinoma cell line HeLa were obtained from the ATCC. Burkitt’s lymphoma cell line Raji was kindly provided by M. Ressing (Dept. of Molecular Cell Biology, LUMC). All cell lines were cultured in IMDM with 10% FCS.

### Isolation and culture of non-hematopoietic cells

Fibroblasts, keratinocytes, proximal tubular epithelial cells (PTEC), melanocytes, primary bronchial epithelial cells (PBEC) and human umbilical vein endothelial cells (HUVEC) were derived from tissue biopsies or surgically removed specimen from patients or healthy individuals at the LUMC. Cornea epithelial (Cornea) and stroma cells were harvested from human cadaveric eyes obtained from the EuroCorneabank, (Beverwijk, the Netherlands). Hepatocytes were obtained from Beckton Dickinson (New Jersey, USA) and colon and small intestinal epithelial cells were obtained from epithelial organoid cultures from the Hubrecht Institute for Developmental Biology and Stem Cell Research (University Medical Centre Utrecht, Utrecht, The Netherlands). Bile duct epithelial cells were purchased from ScienCell (Carlsbad, CA, USA). Fibroblasts, keratinocytes, PTEC, melanocytes and HUVEC were also cultured in the presence of IFN-γ (100 IU/ml) for 4 days to mimic inflammation. In addition, two fibroblast samples were cultured with T-cell culture supernatant for 4 days. Isolation and culture of primary cell types from non-hematopoietic tissues was performed as described in S1 Methods.

### Total RNA isolation

Total RNA was isolated with normal and micro scale RNAqueous isolation kits (Ambion, Thermo Fisher Scientific, Waltham, MA, USA) and treated with DNAse I for 30 min at 37°C. RNA clean-up was performed using RNeasy mini kit (Qiagen, Valencia, CA, USA) and the quality of isolated RNA was checked using Agilent RNA 6000 Nano and Pico chips and Agilent Bioanalyzer (Santa Clara, CA, USA). Total RNA was stored at -80°C for gene expression analysis by human HT-12.0 microarrays or quantitative RT-PCR.

### Human HT-12.0 microarrays

Total RNA as stored at -80°C was thawed to amplify and biotinylate cRNA using the TotalPrep RNA amplification kit (Ambion) and T7 Oligo(dT) primer for First Strand cDNA synthesis. After preparation, cRNA samples were hybridized onto Human HT-12.0 Version 3 or Version 4 Expression BeadChips (Illumina, San Diego, CA, USA). Hybridization was performed in the hybridization oven for 17 hours at 58°C. Chips were stained with streptavidin-Cy3 and scanned using a Bead Array 500 GX scanner. Raw data were imported into Genome Studio (all Illumina) for gene expression quantification. Microarray gene expression data were analyzed with R 2.15 [26]. For this analysis, all samples as measured on Illumina HT-12 chips versions 3 and 4 were combined and merged in one dataset containing all probes as included on both chip versions (n = 39,425), of which 28,280 probes (71.7%) are for designated NM transcripts as annotated by RefSeq. Normalization was done in the lumi R package using the variance stabilizing transformation and quantile normalization [27, 28]. The data discussed in this publication have been deposited in NCBI's Gene Expression Omnibus [12] and are accessible through GEO Series accession number GSE76340 (http://www.ncbi.nlm.nih.gov/geo/query/acc.cgi?acc=GSE76340). Probe fluorescence is determined as log-transformed value or as 2 to the power of the log-transformed value. In comparisons of gene expression profiles between two sample groups, the average log-transformed values for both groups were subtracted and the fold increase in probe fluorescence was calculated as 2 to the power of this difference (2^avg1-avg2^). Expression profiles could not be determined for all genes that have been described as (non-)hematopoietic lineage specific markers due to probe absence on the Illumina HT12.0 array or inclusion of probes showing overall non-significant fluorescence.

### Quantitative RT-PCR

Total RNA as stored at -80°C was thawed to generate cDNA using M-MLV Reverse Transcriptase and Oligo(dT) primer. Quantitative RT-PCR (q-PCR) was performed using predesigned Taqman Gene Expression assays (S2 Table) and Universal Master Mix II, no UNG (all Thermo Fisher Scientific, Waltham, MA, USA) and amplification was measured in real-time using LightCycler 480 (Roche, Basel, Switzerland). Data were analyzed using LightCycler 480 software and fit points analyses. Data was normalized using three reference genes: *HMBS* (alias: *PBGD*), *GAPDH* and *ACTB* (alias: *β-actin*). Amplifications started with 10 minutes at 95°C, followed by 45 cycles of 30 seconds for denaturing at 95°C, 30 seconds of annealing at 60°C, and 30 seconds extension at again 60°C. In the regression models, Cp was corrected for a weighted average of Cp values for the three reference genes.

### Statistical methods

The predictability of array expression measurements based on RT-PCR measurements was investigated. A quadratic prediction model was built for a set of 24 genes. Measurements of reference genes *HMBS*, *ACTB* and *GAPDH* were available. Missing data was imputed. Data was measured in two batches and genes are corrected against reference genes measured in the same batch. For unsupervised clustering analysis, we estimated the sample relation with hierarchical clustering (average linkage) as provided in the Bioconductor lumi (v2.10.0) R package [28].

## Results

### Validation of the cell type origin of hematopoietic and non-hematopoietic samples

**Fig 1:**
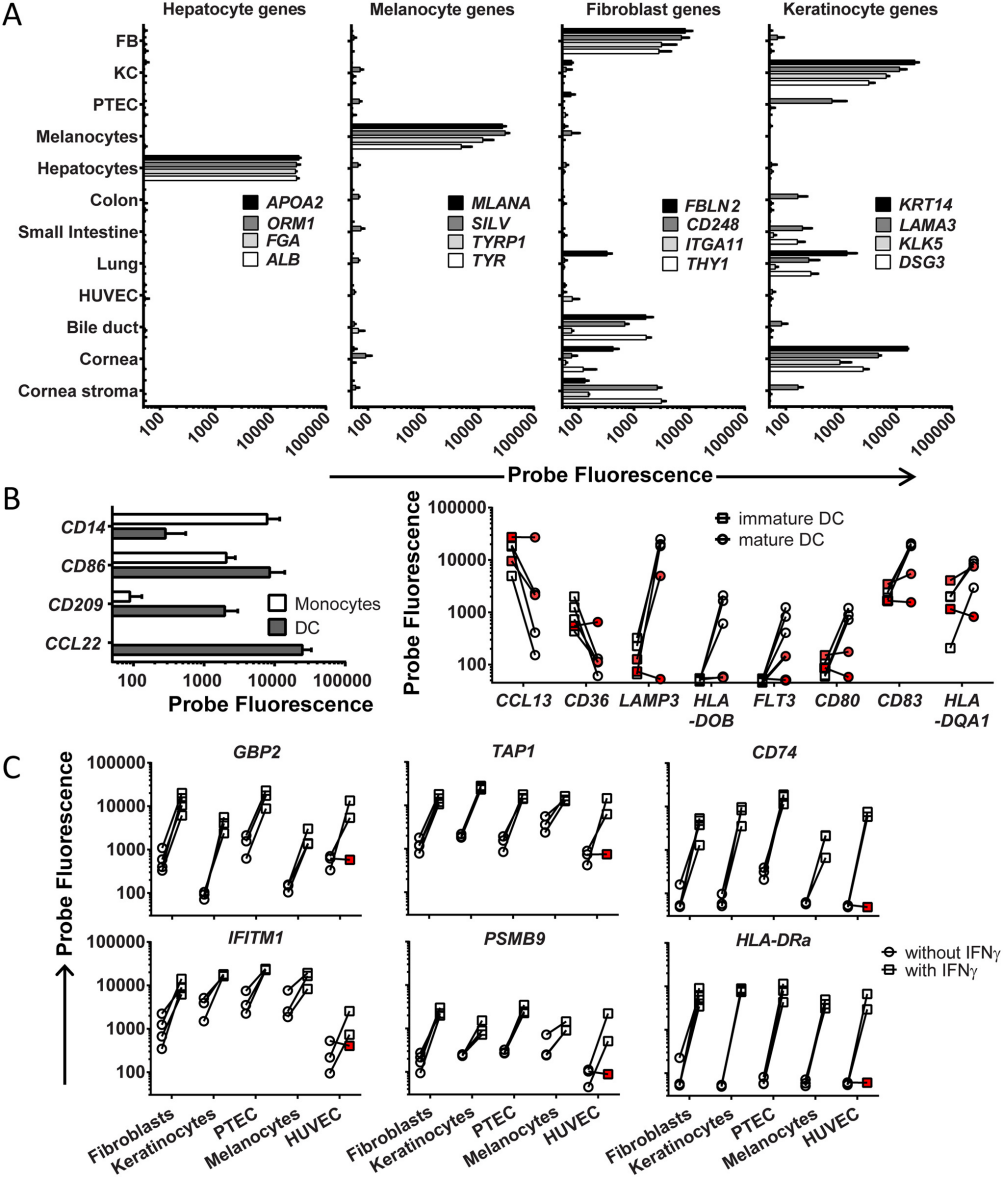
Validation of the cell type origin of (non-)hematopoietic samples. Probe fluorescence as measured by microarray gene expression analysis is depicted on the x-axis in logarithmic scale. (A) Gene expression for various cell type-associated genes as determined by microarray gene expression analysis is shown. Hepatocyte-specific expression is shown for *APOA2*, *ORM1*, *FGA* and *ALB* and melanocyte-specific expression is shown for *MLANA*, *SILV*, *TYRP1* and *TYR*. Fibroblasts-associated expression as defined by detectable expression in fibroblasts as well as a limited number of other non-hematopoietic cell types is shown for *FBLN2*, *CD248*, *ITGA11* and *THY1* and keratinocyte-associated expression is demonstrated for *KRT14*, *LAMA3*, *KLK5* and *DSG3*. Fibroblasts, FB; keratinocytes, KC. (B) Gene expression for monocytes and dendritic cells (DC) is shown (left graph) as well as for immature and mature DC (right graph). Down-regulation of *CD14* and up-regulation of *CD86*, *CD209* and *CCL22* is shown for DC (filled bars) cultured from monocytes (open bars) with GM-CSF and IL-4 (left graph). To validate maturation of DC, down-regulation of markers for immature DC (*CCL13* and *CD36*) and up-regulation of markers for mature DC (*LAMP3*, *HLA-DOB*, *FLT3*, *CD80*, *CD83* and *HLA-DQA1*) was checked (right graph). Immature DC (imDC) are depicted by squares and mature DC (matDC) are indicated by circles. Red symbols indicate samples which have been excluded from the dataset for incomplete maturation. (C) Various non-hematopoietic cell types (fibroblasts, keratinocytes, PTEC, melanocytes and HUVEC) were cultured in the presence of IFN-γ (100 IU/ml) for 4 days to mimic inflammation. Expression of various genes that are known to be induced by IFN-γ is shown (*GBP2*, *IFITM1*), including genes that are involved in HLA processing and presentation (*TAP1*, *CD74*, *PSMB9* and *HLA-DRA*). Circles represent samples cultured without IFN-γ, while squares indicate samples after IFN-γ treatment. Red symbols indicate samples which have been excluded from the dataset for incomplete maturation.

Various hematological malignancies of different origins and their healthy non-malignant counterparts as well as various cell types isolated or cultured from non-hematopoietic tissues were collected for gene expression profiling by Illumina HT12.0 microarrays to enable high-throughput analysis and selection of targets with relevant expression profiles for immunotherapy of hematological malignancies. The majority of hematopoietic cell types have been included directly after isolation by flow cytometry based on expression of specific surface markers. The genes for isolation markers CD19, CD3, CD14 and CD34 showed restricted expression to B cells, T cells, monocytes and HSC, respectively, thereby confirming the origin of hematopoietic cell samples (data not shown). All cell types from healthy non-hematopoietic tissues were included after short-term incubation or culture under specific conditions to eliminate contaminating peripheral blood cells. These cell types were checked and negative for expression of hematopoietic marker genes, indicating that contamination with peripheral blood cells was below the detection limit (data not shown). In addition, we generated expression profiles for various known cell type-associated genes to confirm the origin of non-hematopoietic cell samples in our dataset. In Fig 1A, expression of cell type-specific or -associated genes are shown for hepatocytes, melanocytes, fibroblasts and keratinocytes. Expression profiles for cell type-associated genes for PTEC, HUVEC and PBEC (lung) are depicted in S1 Fig. In addition, profiles for genes that are expressed in the gut are shown as well as profiles for genes with more specific expression in the small intestine. For bile duct epithelial cells, cell type-associated genes with significant probe fluorescence could not be identified and cornea epithelial and cornea stroma cells were shown to share significant gene expression with keratinocytes and fibroblasts, respectively (Fig 1).

For *in vitro* modified hematopoietic cells, we checked whether the induced cell types properly differentiated from their original cell type based on expression of a number of pre-defined genes. DC cultured from monocytes with GM-CSF and IL-4 were checked for down-regulation of the monocyte-specific *CD14* gene and up-regulation of DC-specific genes (Fig 1B, left panel). Full DC maturation was confirmed by induced gene expression of known maturation markers and down-regulated expression of markers for immature DC (Fig 1B, right panel). The combination of these markers allows for quality control and two DC samples were excluded from the dataset for no or incomplete maturation (Fig 1B). Macrophages (MΦ) were generated from isolated monocytes by culturing with GM-CSF (type I MΦ) or M-CSF (type II MΦ). Differentiation to macrophages was confirmed by induced expression of *MMP9* [29] and *SPP1* [30] and expression of *CCL2* [31] and *CD163* [32] was stronger in type II than in type I MΦ (data not shown).

To evaluate the role of inflammation on gene expression in non-hematopoietic cells, we cultured various non-hematopoietic cell types (fibroblasts, keratinocytes, PTEC, melanocytes and HUVEC) in the presence of IFN-γ. The effect of IFN-γ was checked by measuring expression of interferon-inducible genes as well as genes involved in HLA-class I and II processing and presentation (Fig 1C). All samples showed strong up-regulation of these genes after IFN-γ treatment, except for one HUVEC sample (HUVEC #3), which was excluded from the dataset.

As additional unbiased method for cell type validation, we performed hierarchical clustering for the complete panel of healthy (non-)hematopoietic samples based on expression profiling of all genes (S2 Fig). In this analysis, hematopoietic cell types were distinguished from non-hematopoietic cell types and samples with the same cell type origin clustered based on shared gene expression profiles. This further validated the cell type origin of the (non-)hematopoietic cell samples in the microarray dataset and confirmed lack of detectable contamination of non-hematopoietic cell samples with peripheral blood cells.

### Inclusion of hematological malignancies of different origins

All malignant hematopoietic cell types were categorized according to the WHO classification standards and analyzed for cytogenetic abnormalities and, if applicable, for morphology and immune phenotype (Table 1 and complex karyotypes in S1 Table). A wide variety of samples were included to obtain a broad repertoire of hematological malignancies. Malignant cell populations were isolated by flow cytometry based on expression of specific surface markers. Gene expression for these surface markers is depicted in S3 Fig. In addition to primary hematological malignancies, we included tumor cell lines K562, T2, AML-193, THP-1, Daudi, Raji, Jurkat and HeLa.

**Table 1:** Characteristics of leukemic cells selected for microarray gene expression analysis.

**Sample**^a^	**Origin**^b^	**WHO classification**^c^				**Other genetic abnormalities**^d^			
AML 2467 (CD33/CD14)	PB	Acute myeloid leukemia with inv(16)(p13.1q22); *CBFB-MYH11*				trisomy 8, trisomy 14, trisomy 21			
AML 1310 (CD33/CD14)	PB	Acute myeloid leukemia with inv(16)(p13.1q22); *CBFB-MYH11*				-			
AML 3097 (CD33)	BM	Acute promyelocytic leukemia with t(15;17)(q22;q12); *PML-RARA*				*CEBPA* polymorphism (6bp insertion)^e^			
AML 2179 (CD33)	PB	Acute promyelocytic leukemia with t(15;17)(q22;q12); *PML-RARA*				-			
AML 1591 (CD33)	PB	Acute promyelocytic leukemia with t(15;17)(q22;q12); *PML-RARA*				*FLT3*-ITD positive			
AML 1466 (CD33/CD14)	BM	Acute myeloid leukemia with t(9;11) (p22q23); *MLLT3-MLL*				-			
AML 4781 (CD33/CD14)	PB	Acute myeloid leukemia with inv(3)(q21q26.2); *RPN1-EVI1*				*EVI1* overexpression, monosomy 7, del (7) (q21), add(12) (p11); complex^f^			
AML 587 (CD33)	BM	Acute myeloid leukemia with mutated *NPM1*				*FLT3*-ITD positive			
AML 2536 (CD33)	BM	Acute myeloid leukemia with mutated *NPM1*				-			
AML 4443 (CD33)	PB	Acute myeloid leukemia with mutated *NPM1*				*FLT3*-ITD positive			
AML 6395 (CD33)	PB	Acute myeloid leukemia with mutated *NPM1*				-			
AML 3370 (CD33)	PB	Acute myeloid leukemia with mutated *NPM1*				-			
AML 5205 (CD33/CD14)	PB	Acute myeloid leukemia with mutated *NPM1*				*FLT3*-ITD positive			
AML 3590 (CD33)	PB	t-AML				-			
AML 3714 (CD33)	BM	AML-NOS, with minimal differentiation				complex^f^			
AML 5596 (CD33)	BM	AML-NOS, without maturation				-			
AML 3778 (CD33)	PB	AML-NOS, without maturation				complex^f^			
AML 3870 (CD33)	BM	AML-NOS, with maturation				trisomy 8			
AML 6283 (CD33)	BM	AML-NOS, with maturation				*FLT3*-ITD positive			
AML 1143 (CD33)	PB	AML-NOS, acute monoblastic leukemia				-			
AML 3009 (CD33)	BM	AML-NOS, acute monoblastic leukemia				*FLT3*-ITD positive			
AML 5074 (CD33/CD14)	PB	AML-NOS, acute monoblastic leukemia				complex^f^			
**Sample**	**origin**	**WHO classification**				**other genetic abnormalities**			
ALL 2391 (CD19)	BM	B Lymphoblastic leukemia NOS				trisomy 5			
ALL 2872 (CD19)	BM	B Lymphoblastic leukemia NOS				-			
ALL 1299 (CD19)	BM	B Lymphoblastic leukemia NOS				del(6) (q21q23), del(Y)			
ALL 3281 (CD19)	PB	B Lymphoblastic leukemia NOS				-			
ALL 5903 (CD19)	BM	B Lymphoblastic leukemia with t(9;22)(q34;q11.2); *BCR-ABL1* p190				complex^f^			
ALL 3639 (CD19)	BM	B Lymphoblastic leukemia with t(9;22)(q34;q11.2); *BCR-ABL1* p190				trisomy 5, trisomy 8			
ALL 2375 (CD19)	BM	B Lymphoblastic leukemia with t(4;11) (q21;q23); *MLL* rearranged				-			
ALL 1833 (CD19)	PB	B Lymphoblastic leukemia with t(11;19) (q23;p13.3); *MLL* rearranged				-			
ALL 3655 (CD19)	BM	B Lymphoblastic leukemia with hyperdiploidy				complex^f^			
**Sample**	**origin**	**WHO classification**				**Other genetic abnormalities**			
CML 3471 (CD34)^g^	BM	Chronic myelogenous leukemia, *BCR-ABL1* positive				del (9q); complex^f^			
CML 5036 (CD34)^h^	PB	Chronic myelogenous leukemia, *BCR-ABL1* positive				t(2;9;22) (p13;q34;q11)			
CML 3087 (CD34)^h^	BM	Chronic myelogenous leukemia, *BCR-ABL1* positive				del (Xp), del (17q); complex^f^			
CML 4779 (CD34)^h^	BM	Chronic myelogenous leukemia, *BCR-ABL1* positive				-			
CML 1303 (CD34)^h^	BM	Chronic myelogenous leukemia, *BCR-ABL1* positive				-			
**Sample**	**origin**	**WHO classification**		**Genetic abnormalities**^i^					
				**Trisomy 12**		**13q14.3**		***ATM***	***p53***
CLL 1695 (CD19/CD5)	BM	Chronic lymphocytic leukemia		absent		del/del^j^		del	normal
CLL 4725 (CD19/CD5)^f^	BM	Chronic lymphocytic leukemia		absent		del		normal	del
CLL 5535 (CD19/CD5)	PB	Chronic lymphocytic leukemia		absent		del		normal	normal
CLL 6242 (CD19/CD5)	PB	Chronic lymphocytic leukemia		absent		normal		normal	del
CLL 2159 (CD19/CD5)	PB	Chronic lymphocytic leukemia		absent		del/del^j^		normal	normal
**Sample**	**origin**	**WHO classification**	**Genetic abnormalities**^i^						
			**Hyperdiploidy**	**numeric abnorm**	**chr1**	**13q14**	**p53**	**IgH rearrangements**	**t(14;16) (q32;q23)**
MM 5987 (CD38)^f^	BM	Plasma cell myeloma	hyperdiploid	+9, +15	+1q	del	normal	t(4;14)(p16;q32)	absent
MM 5744 (CD38)^f^	BM	Plasma cell myeloma	diploid	absent	+1q	del	normal	t(4;14)(p16;q32)	absent
MM 6622 (CD38)	BM	Plasma cell myeloma	diploid	absent	normal	normal	normal	normal	absent
MM 4298 (CD38)	BM	Plasma cell myeloma	diploid	-15	normal	del	del	t(4;14)(p16;q32)	absent
MM 5019 (CD38)^f^	BM	Plasma cell myeloma	diploid	absent	normal	del	normal	t(11;14)(q13;q32)	absent

^a^ Markers for flow cytometric isolation of malignant cells are indicated between brackets. AML cells have been isolated by expression of CD33 (CD33) or a combination of CD33 and CD14 (CD33/CD14) in which CD33 expressing AML cells positive or negative for monocyte lineage differentiation marker CD14 have been selected as two separate cell populations. CLL cells have been selected for co-expression of CD19 and CD5. ALL, CML and MM cells have been isolated by expression of CD19, CD34 and CD38, respectively.

^b^ Origin of sample either peripheral blood (PB) or bone marrow (BM) mononuclear cells.

^c^ WHO classification as described by Swerdlow et al, 2008.

^d^ Other abnormalities include numerical abnormalities, CEBPA mutations, FLT3-ITD, or EVI1 overexpression.

^e^ Partial del(6q?) in fraction of the cells.

^f^ Complex karyotypes are depicted in S1 Table.

^g^ CML in blast crisis.

^h^ CML in chronic phase.

^i^ Genetic abnormalities as detected by FISH for CLL or MM.

^j^ del/del; homozygous deletion.

### Validation of microarray gene expression analysis by quantitative RT-PCR

For all 39,426 probes as included in HT12.0 microarray versions 3 and 4, we determined the maximum probe fluorescence as measured in any of the 166 (non-)hematopoietic cell samples in the dataset. Maximum probe fluorescence showed great variability, ranging from log 5.6 to log 15.2. No or low fluorescence can be the consequence of poor probe quality or absence or low expression of the gene transcript in the dataset, while high probe fluorescence indicates that the gene transcript is strongly expressed. To investigate whether gene expression patterns can be accurately and reliably established with our microarray dataset, we validated microarray gene expression data by q-PCR for 24 genes that were selected for different maximum probe fluorescence values.

For 53 samples, which were selected based on wide variability in gene expression throughout the 24 genes, cDNA was generated from the same mRNA source as used for microarray gene expression analysis and gene expression was measured by q-PCR. The complete set of samples, genes, probes and Taqman assays are depicted in S2 and S3 Tables. Using a quadratic prediction model, the correlation between microarray and q-PCR expression data was determined by calculating the coefficient of determination (R^2^). The average correlation for the entire set of 24 genes was strong (corrected R^2^ = 0.868). Correlation plots for separate genes are depicted in S4 Fig. In Table 2, individual R^2^ values are depicted as well as the maximum probe fluorescence as measured by microarray gene expression analysis in the sample set for q-PCR validation. Table 2 shows that R^2^ > 0.67, which indicates that the variability in gene expression in more than two third of the samples fits between microarray and q-PCR analysis, was measured for all 11 genes (100%) with a maximum probe fluorescence > log 11, whereas for the remaining 13 genes with maximum probe fluorescence < log 11, only 3 genes (23%) showed R^2^ > 0.67.

**Table 2:** R^2^ values for regression analysis between microarray and q-PCR^a^.

Gene^b^	Probe 1^c^	Probe 2^c^	Maximum expression in dataset^d^			R^2^
			Probe 1	Probe 2	Probe average^e^	
*HLA-DRA*	ILMN_1689655	ILMN_2157441	15.03	14.61	14.82	**0.925**^f^
*TAP1*	ILMN_1751079		14.78			**0.759**
*COL1A1*	ILMN_1701308		14.75			**0.948**
*CD14*	ILMN_1740015	ILMN_2396444	13.20	14.80	14.00	**0.732**
*ELANE*	ILMN_1706635		13.87			**0.783**
*CD34*	ILMN_1732799	ILMN_2341229	13.59	13.54	13.57	**0.944**
*CD19*	ILMN_1782704		13.49			**0.985**
*PRTN3*	ILMN_1753584		13.16			**0.867**
*CD33*	ILMN_1747622		11.66			**0.834**
*KRT8*	ILMN_1753584		11.55			**0.974**
*P2RX5*	ILMN_1677793		11.09			**0.679**
*MOB3A*	ILMN_1721344		10.88			0.326
*POLE*	ILMN_1728199		10.76			0.409
*C19orf48*	ILMN_1759184	ILMN_2383484	11.34	10.11	10.73	0.266
*HMHA1*	ILMN_1811392		10.11			**0.779**
*CLYBL*	ILMN_1663538		9.30			0.487
*ROR1*	ILMN_1655904		9.20			**0.938**
*COIL*	ILMN_1688034		8.65			-0.001
*MS4A1*^g^	ILMN_1776939		8.62			0.320
*PFAS*	ILMN_1755862		8.61			0.216
*PTPRC*^h^	ILMN_2340217	ILMN_1653652	8.07	8.78	8.43	0.347
*APOBEC3B*	ILMN_2219466		8.23			0.652
*TTK*	ILMN_1788166		8.09			0.545
*WT1*	ILMN_1802174		8.00			**0.856**

^a^ R^2^ values for individual genes were calculated from the regression model.

^b^ Official gene symbols are depicted for genes as included in the q-PCR validation set.

^c^ Illumina probe IDs as measured in the microarray database.

^d^ Maximum probe fluorescence as measured by microarray gene expression analysis in the sample set for q-PCR validation.

^e^ Average of maximum probe fluorescence is given for genes with more than 1 probe.

^f^ R^2^ >0.667 are bold.

^g^ MS4A1 is also known as CD20.

^h^ PTPRC is also known as CD45.

In conclusion, our data show that gene expression patterns can be accurately established with our microarray dataset for transcripts with a maximum probe fluorescence > log 11, while q-PCR analysis is recommended if the transcript of interest has a maximum probe fluorescence < log 11. If maximum probe fluorescence is < log 11, less detailed qualitative analysis can still be performed, but quantitative analysis by microarray gene expression is not possible.

### The effect of inflammation on gene expression in non-hematopoietic cell types

**Fig 2:**
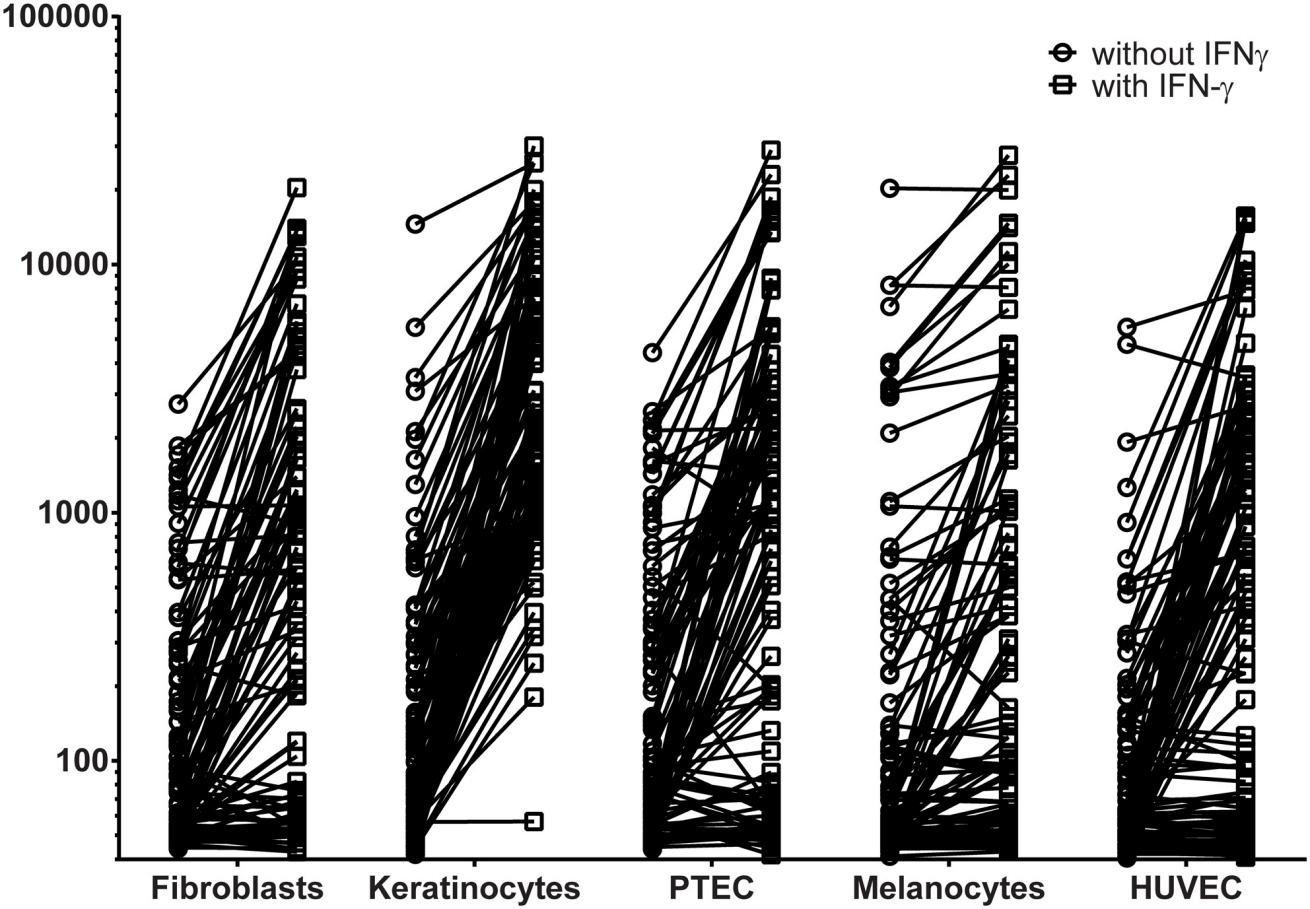
Effect of IFN-γ on gene expression in non-hematopoietic cell types. Microarray gene expression analysis was performed on fibroblasts, keratinocytes, PTEC, melanocytes and HUVEC cultured in the presence of IFN-γ and probe fluorescence was compared with the same cell samples cultured in the absence of IFN-γ. All probes that were >10-fold up-regulated after IFN-γ treatment in one or more cell types are depicted. Probe fluorescence is shown on the y-axis in logarithmic scale. Circles show gene expression in the absence of IFN-γ, while squares indicate expression in the presence of IFN-γ. For each cell type, the number of probes that are >10-fold up-regulated by IFN-γ is shown.

To evaluate gene expression in non-hematopoietic cell types under inflammatory conditions, we performed microarray gene expression analysis on fibroblasts, keratinocytes, PTEC, melanocytes and HUVEC cultured in the presence of IFN-γ and compared probe fluorescence with the same samples that were cultured in the absence of cytokines. In total, 9 probes were >10-fold induced by IFN-γ in all 5 non-hematopoietic cell types. These probes were specific for interferon-inducible genes (*GBP2*) as well as genes involved in HLA-class II processing and presentation (*CD74*, *HLA-DMA*, *HLA-DPA1*, *HLA-DRA*, *HLA-DRB4* and *LOC649143*). In addition, 106 probes were > 10-fold up-regulated in at least one non-hematopoietic cell type (Fig 2 and S4 Table). Up-regulated gene expression by IFN-γ was detected for 32, 83, 35, 11 and 40 probes in fibroblasts, keratinocytes, PTEC, melanocytes and HUVEC, respectively, while expression levels in the absence of IFN-γ were similar between these non-hematopoietic cell types.

To determine whether gene expression patterns are different between cell types cultured with a cocktail of inflammatory cytokines as compared to IFN-γ alone, we performed microarray gene expression analysis on fibroblast samples that we cultured in the presence of culture supernatant from an activated CD4 T-cell clone in which high levels of IFN-γ, IL-13, TNF-α and IL-2 were detected. Table 3 shows all separate probes (n = 47) for genes that are >10-fold up-regulated by IFN-γ or T-cell supernatant. The data show that 46 out of 47 probes were at least 5-fold up-regulated under both conditions, indicating that IFN-γ can be used as single agent to induce an inflammatory gene expression signature.

**Table 3:** Gene expression in skin fibroblasts as induced by IFN-γ and T-cell supernatant.

Probe ID^a^	Gene^b^	Fibroblasts		Fibroblasts + IFN-γ		Fold Increase^d^	Fibroblasts		Fibroblasts + T-sup^e^		Fold Increase
		AVG^c^	SD^c^	AVG	SD		AVG	SD	AVG	SD	
ILMN_1705247	*ACSL5*	71,48	18,18	915,69	4,61	**12,81**	57,07	5,64	410,79	21,92	*7*,*20*
ILMN_1756862	*APOL3*	127,45	50,12	1744,54	88,76	**13,69**	126,02	3,27	1215,58	198,99	*9*,*65*
ILMN_1720048	*CCL2*	405,52	320,65	566,98	379,69	1,40	146,88	58,76	3739,10	578,67	**25,46**
ILMN_2098126	*CCL5*	56,56	5,03	87,02	7,13	1,54	80,04	4,21	1448,07	17,78	**18,09**
ILMN_1773352	*CCL5*	55,04	4,49	70,60	14,72	1,28	52,54	0,90	564,89	13,32	**10,75**
ILMN_1772964	*CCL8*	79,79	26,74	654,98	396,22	*8*,*21*	76,86	23,08	1092,76	27,37	**14,22**
ILMN_1736567	*CD74*	105,73	53,25	4540,97	791,32	**42,95**	59,62	4,58	6697,04	438,61	**112,33**
ILMN_2379644	*CD74*	77,73	31,38	1627,96	282,95	**20,94**	46,04	4,27	3008,86	137,93	**65,35**
ILMN_1761464	*CD74*	51,45	1,29	730,79	41,60	**14,20**	45,18	2,03	518,30	79,50	**11,47**
ILMN_2047511	*CENTA1*	187,61	62,18	1916,52	1491,98	**10,22**	94,46	27,37	664,84	489,58	*7*,*04*
ILMN_1791759	*CXCL10*	51,04	4,63	178,24	64,32	3,49	54,72	1,54	1600,24	844,80	**29,24**
ILMN_1745356	*CXCL9*	48,55	4,44	303,32	131,99	*6*,*25*	41,91	1,69	5149,88	3359,40	**122,89**
ILMN_2388547	*EPSTI1*	124,13	41,46	1691,41	606,22	**13,63**	165,03	48,22	2084,78	643,31	**12,63**
ILMN_1701114	*GBP1*	183,62	52,59	2569,15	182,46	**13,99**	182,31	43,53	3793,63	414,77	**20,81**
ILMN_2148785	*GBP1*	143,83	37,33	1191,59	6,56	*8*,*28*	274,42	56,92	5802,20	1534,56	**21,14**
ILMN_1774077	*GBP2*	745,66	345,68	17626,86	2385,66	**23,64**	582,62	90,58	7100,17	300,24	**12,19**
ILMN_1771385	*GBP4*	48,49	2,67	903,78	21,36	**18,64**	57,47	0,85	1439,61	406,66	**25,05**
ILMN_2114568	*GBP5*	56,21	4,33	1124,80	148,70	**20,01**	48,52	1,19	1189,30	25,32	**24,51**
ILMN_1803945	*HCP5*	91,78	15,97	892,47	51,92	*9*,*72*	71,62	1,81	936,66	97,71	**13,08**
ILMN_1778401	*HLA-B*	1305,90	484,58	9930,80	649,29	*7*,*60*	1354,92	495,95	15916,89	2069,86	**11,75**
ILMN_1695311	*HLA-DMA*	542,10	87,50	7641,55	2580,39	**14,10**	355,31	31,05	3309,74	1393,47	*9*,*32*
ILMN_1761733	*HLA-DMB*	89,92	32,72	2127,14	1065,96	**23,66**	66,70	10,93	930,50	471,41	**13,95**
ILMN_1659075	*HLA-DOA*	51,01	4,51	897,08	186,07	**17,59**	51,14	1,29	312,10	118,38	*6*,*10*
ILMN_1772218	*HLA-DPA1*	160,29	7,45	3916,33	585,53	**24,43**	119,74	3,57	2708,72	724,98	**22,62**
ILMN_1808405	*HLA-DQA1*	59,19	9,86	887,82	661,25	**15,00**	41,27	0,96	870,79	606,77	**21,10**
ILMN_1689655	*HLA-DRA*	176,76	126,36	12566,25	3920,68	**71,09**	93,09	0,69	4449,93	1823,37	**47,80**
ILMN_2157441	*HLA-DRA*	138,34	86,70	5530,45	687,54	**39,98**	62,36	1,05	5817,44	2067,78	**93,29**
ILMN_1715169	*HLA-DRB1*	49,64	3,33	302,36	253,61	*6*,*09*	51,16	0,40	880,52	833,29	**17,21**
ILMN_1752592	*HLA-DRB4*	97,08	25,57	3135,67	1486,29	**32,30**	59,02	1,21	2467,91	384,34	**41,81**
ILMN_1697499	*HLA-DRB5*	73,82	26,82	826,23	777,15	**11,19**	48,12	0,54	1122,44	1073,92	**23,32**
ILMN_2066060	*HLA-DRB6*	56,77	0,82	317,56	109,15	*5*,*59*	60,67	1,64	812,22	438,83	**13,39**
ILMN_2066066	*HLA-DRB6*	52,33	1,69	192,74	48,32	3,68	48,40	2,49	988,46	215,83	**20,42**
ILMN_1723912	*IFI44L*	63,42	5,51	1192,97	174,91	**18,81**	64,62	10,67	1230,95	168,61	**19,05**
ILMN_2347798	*IFI6*	212,00	45,36	2234,49	782,87	**10,54**	664,54	215,55	12733,61	8737,71	**19,16**
ILMN_1739428	*IFIT2*	148,34	6,69	2190,73	666,22	**14,77**	333,39	47,93	2205,74	1097,87	*6*,*62*
ILMN_1701789	*IFIT3*	309,27	24,42	5293,10	106,34	**17,11**	248,64	35,15	2465,82	954,09	*9*,*92*
ILMN_1664543	*IFIT3*	61,94	6,84	500,90	52,90	*8*,*09*	111,53	4,69	1162,56	556,31	**10,42**
ILMN_2334296	*IL18BP*	124,90	13,66	3132,98	608,04	**25,08**	68,50	2,03	2266,51	1379,17	**33,09**
ILMN_2368530	*IL32*	54,42	6,19	149,61	4,61	2,75	109,67	4,68	1873,74	978,51	**17,09**
ILMN_1656310	*INDO*	48,99	9,14	523,11	154,06	**10,68**	45,64	0,65	5685,52	4062,65	**124,58**
ILMN_1708375	*IRF1*	498,42	212,95	2664,97	295,19	*5*,*35*	277,69	29,54	4185,18	822,05	**15,07**
ILMN_1662358	*MX1*	198,75	6,86	10822,34	4316,73	**54,45**	671,64	353,82	6055,89	2475,95	*9*,*02*
ILMN_1701613	*RARRES3*	291,17	134,16	9431,88	2494,20	**32,39**	190,98	15,69	4008,61	1773,14	**20,99**
ILMN_1751079	*TAP1*	1184,40	16,21	13220,08	1203,62	**11,16**	824,61	168,64	6181,11	527,53	*7*,*50*
ILMN_1678841	*UBD*	49,97	2,18	76,70	15,87	1,53	52,95	1,42	1077,09	664,73	**20,34**
ILMN_1727271	*WARS*	1219,84	167,93	18566,63	8233,61	**15,22**	364,88	11,13	7669,97	593,84	**21,02**
ILMN_2337655	*WARS*	1267,64	209,92	13887,52	4384,17	**10,96**	508,79	30,61	11333,68	2227,47	**22,28**

^**a**^ Illumina probe IDs as included in the microarray database.

^b^ Official gene symbols are depicted for genes that are >10-fold up-regulated by IFN-γ, T-cell supernatant or both.

^c^ AVG and SD are the average and standard deviation of probe fluorescence as measured in different fibroblast samples, respectively.

^d^ Fold increase was calculated from the average probe fluorescence as measured in fibroblasts after pre-treatment with IFN-γ or T-cell supernatant divided by the average probe fluorescence in the corresponding fibroblast samples cultured without cytokines. Values in bold indicate >10-fold upregulated gene expression by IFN-γ, T-cell supernatant or both.

^e^ T-sup; T-cell supernatant harvested from an activated CD4 T-cell clone containing high levels of inflammatory cytokines.

### Gene expression profiles of immunotherapeutic targets for hematological malignancies

**Fig 3:**
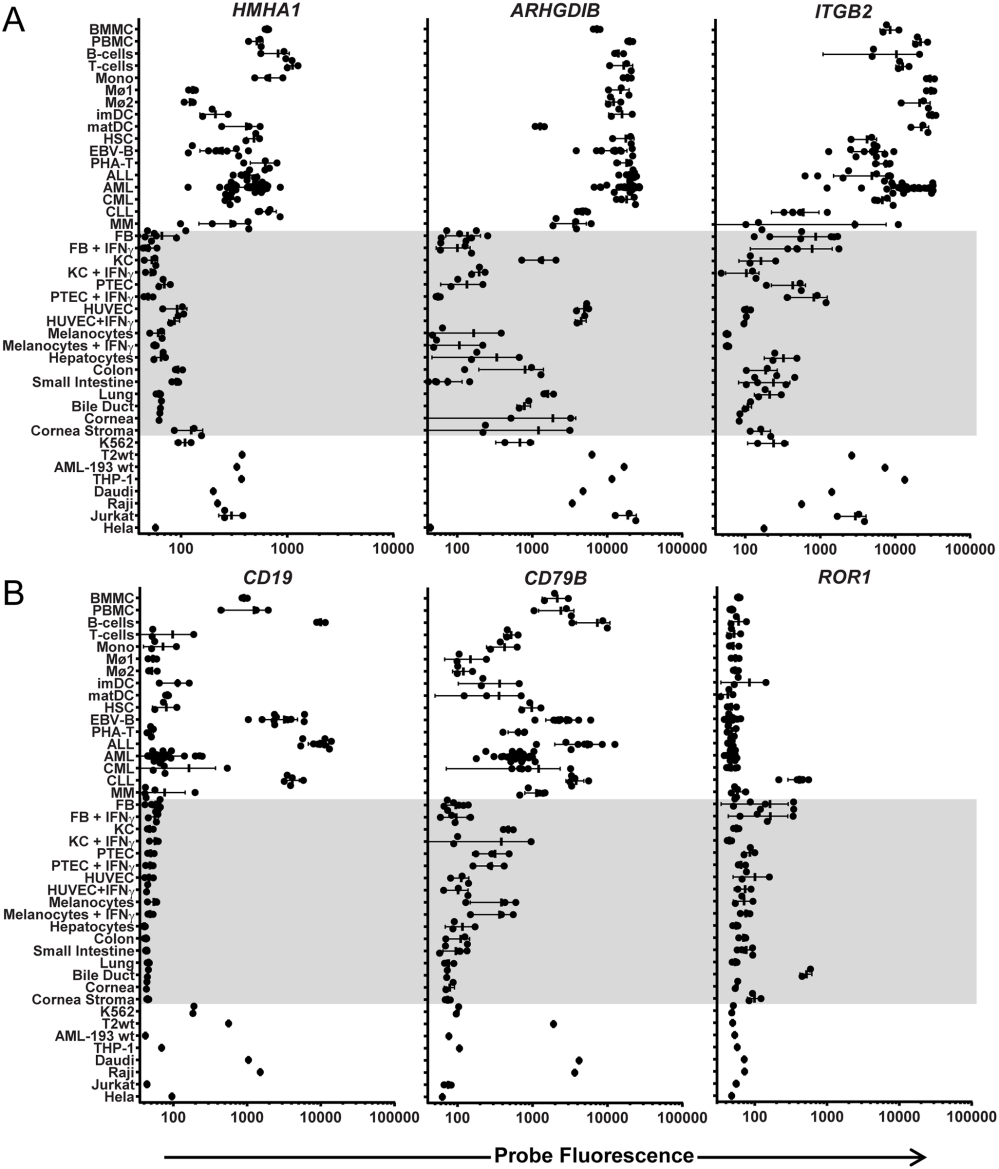
Gene expression profiles for potential targets for immunotherapy of hematological malignancies. Gene expression profiles were generated for hematopoiesis-restricted minor histocompatibility antigens and B-cell specific surface antigens as potential targets for immunotherapy of hematological malignancies. (A) Gene expression profiles for minor histocompatibility antigens HA-1 (*HMHA1*), LB-ARHGDIB-1R (*ARHGDIB*) and LB-ITGB2-1 (*ITGB2*). (B) Gene expression profiles for B-cell specific antigens *CD19*, *CD79B* and *ROR1*. Probe fluorescence intensity is shown on the x-axis in logarithmic scale. On the y-axis malignant and healthy (non-)hematopoietic cell types as included in the microarray dataset are shown. Each dot represents a different sample and the mean and standard deviation of gene expression is shown for each cell type.

Minor histocompatibility antigens with hematopoiesis-restricted expression are relevant targets to selectively induce anti-tumor reactivity after alloSCT. Only a limited number of minor histocompatibility antigens have been reported to be hematopoiesis-restricted. To evaluate expression of these antigens in our microarray dataset of malignant and healthy hematopoietic and non-hematopoietic cell types, we generated gene expression profiles for the known therapeutic minor histocompatibility antigens HA-1 (*HMHA1*) [33, 34], LB-ARHGDIB-1R (*ARHGDIB*) [35, 36] and LB-ITGB2-1 (*ITGB2*) [37]. Fig 3A shows that maximum probe fluorescence was < log 11 for *HMHA1*, while values > log 11 were measured for *ARHGDIB* and *ITGB2*. Despite low probe fluorescence, *HMHA1* was accurately measured by microarray gene expression analysis as illustrated by strong association with q-PCR data (Table 2; R^2^ = 0.779). The data also show that expression of *HMHA1* and *ARHGDIB* was restricted or predominant in all or the majority of hematopoietic cells, while expression of *ITGB2* was specific for certain hematopoietic lineages. No expression of *HMHA1* could be measured in any of the non-hematopoietic cell types even when cultured with IFN-γ, while *ARHGDIB* and *ITGB2* showed intermediate and low expression in HUVEC and fibroblasts, respectively. In conclusion, microarray analysis confirmed restricted or predominant gene expression in hematopoietic cells for therapeutic minor histocompatibility antigens.

In addition to minor histocompatibility antigens, surface antigens with restricted expression on hematopoietic lineages or malignancies are relevant targets for therapeutic antibodies or CAR T-cell therapy. We therefore explored our microarray dataset to evaluate gene expression for surface antigens with known expression on B cells (*CD19* and *CD79B*) or B-cell malignancies (*ROR1*). Fig 3B shows that maximum probe fluorescence was > log 11 for *CD19* and *CD79B*, while values < log 11 were observed for *ROR1*. Despite low probe fluorescence, *ROR1* microarray data were reliable as confirmed by q-PCR data (R^2^ = 0.938, Table 2). Furthermore, the data show that *CD19* was highly expressed in healthy B cells, ALL and CLL, while no expression was detected in any other (non-)hematopoietic cell type. *ROR1* demonstrated overexpression in CLL, while expression of this gene was not detectable in healthy B cells. However, *ROR1* expression was also found in biliary epithelial cells and to variable extents in skin fibroblasts. Finally, expression of *CD79B* was most pronounced in the B-cell lineage, but expression was also found in a variety of other (non-)hematopoietic cell types. Thus, restricted or predominant expression in (malignant) B cells could be confirmed by microarray gene expression analysis for surface antigens with known B-cell specific expression.

In conclusion, the data show that our microarray gene expression dataset as collected for (malignant) hematopoietic cell samples and non-hematopoietic cell types cultured under steady state and inflammatory conditions provides a high-throughput platform for detailed analysis and selection of candidate targets with hematopoiesis (lineage)-restricted expression for immunotherapy of hematological malignancies.

## Discussion

In this study, we performed microarray gene expression analysis on malignant and healthy hematopoietic cells and various non-hematopoietic cell types cultured under steady state and inflammatory conditions. Quality control was performed to confirm cell-type origin of the samples and to exclude peripheral blood contamination of non-hematopoietic samples. Validation of gene expression was performed by quantitative RT-PCR and an inflammatory gene signature was established by comparing gene expression between different non-hematopoietic cells after pre-treatment with IFN-γ. Furthermore, we demonstrated the value of the microarray dataset to generate gene expression profiles for potential targets for immunotherapy of hematological malignancies.

Validation of the Illumina HT12 microarray gene expression dataset by q-PCR analysis demonstrated a strong correlation between both platforms with an overall corrected R^2^ of 0.868. However, maximum probe fluorescence as measured in any cell type of the dataset varied significantly. No or low fluorescence can be the consequence of poor probe quality or absence or low expression of the gene transcript in the dataset, while high probe fluorescence indicates that the gene transcript is strongly expressed. Strong correlation between q-PCR and microarray data with R^2^ > 0.667 was obtained for all probes with maximum fluorescence > log 11. Of the 28,280 probes (20,215 when selecting probes for unique genes) for designated NM transcripts on Illumina HT12 chips, fluorescence > log 11 was measured for 4301 probes (15%), 3787 (19%) after selection of probes for unique genes. Provided that ~50% of all genes as present in the human genome are expressed in differentiated cell types [38], expression profiles can be determined with high accuracy for ~40% of genes. For probes with maximum fluorescence < log 11, qualitative analysis can still be performed, but q-PCR studies are recommended for quantitative gene expression analysis.

To evaluate up-regulated gene expression under inflammatory conditions, we cultured various non-hematopoietic cell types in the presence of IFN-γ and compared gene expression to the same cells cultured in the absence of cytokines. In total 106 probes were >10-fold up-regulated in at least one non-hematopoietic cell type with melanocytes being less sensitive to IFN-γ pre-treatment (n = 11 probes) than keratinocytes (n = 83 probes). To determine whether IFN-γ can be used as single agent to mimic inflammation, we also cultured fibroblasts in the presence of T-cell culture supernatant containing high levels of inflammatory cytokines (IFN-γ, IL-13, TNF-α and IL-2). There was great overlap between genes up-regulated by IFN-γ and T-cell culture supernatant, illustrating that IFN-γ as single compound can create an inflammatory gene signature. As such, gene expression analysis of samples cultured with IFN-γ can be used to estimate toxicity of immunotherapeutic targets against non-hematopoietic cells under inflammatory conditions.

In our microarray dataset, contamination of non-hematopoietic cells with peripheral blood cells was excluded to allow identification and selection of genes with hematopoietic (lineage)-restricted expression, which may encode relevant targets for immunotherapy of hematological malignancies. To evaluate the value of our microarray dataset to analyze and select potential targets for immunotherapy of hematological malignancies, we generated gene expression profiles for hematopoiesis-restricted minor histocompatibility antigens that are recognized by specific T cells in the context of HLA. Microarray analysis confirmed restricted or predominant expression of these antigens in hematopoietic cells, but *ARHGDIB* and *ITGB2* also showed intermediate and low expression in endothelial cells and fibroblasts, respectively. Since actual antigen presentation by HLA is not measured by microarray gene expression analysis, additional experiments are required to further evaluate potential toxicity against non-hematopoietic cell types with detectable expression of the gene of interest. For *ARHGDIB* and *ITGB2*, we measured T-cell reactivity against endothelial cells and fibroblasts, but did not find any evidence for toxicity [35, 37]. In addition to minor histocompatibility antigens, we generated gene expression profiles for surface antigens with known B-cell specific expression that can be targeted independent of HLA by CAR-based immunotherapy. Microarray analysis confirmed restricted or predominant expression of these antigens in (malignant) B cells. However, *CD79B* was also expressed in a variety of other (non-)hematopoietic cell types, which was supported by Jahn et al. [39], who demonstrated that intracellular CD79B peptides are presented by HLA and recognized by specific T cells on other cell types than B cells. Since actual surface expression is not measured by microarray gene expression analysis, additional experiments are required to evaluate whether CD79B is an appropriate target for CAR-based therapy. For *ROR1*, microarray data confirmed overexpression in CLL as compared to healthy B cells, supporting its relevance as therapeutic target. *ROR1* expression was also found in biliary epithelial cells and to variable extents in skin fibroblasts, but no evidence has been found that gene expression in these cell types leads to detectable surface expression as illustrated by the safety of CAR-based therapy targeting ROR1 in nonhuman primates [40, 41].

In summary, we performed microarray gene expression analysis on hematological malignancies of different origins, healthy hematopoietic cells and various (IFN-γ pre-treated) non-hematopoietic cell types and demonstrate that our microarray gene expression database allows detailed analysis and selection of candidate targets with hematopoietic (lineage-)restricted expression for immunotherapy of hematological cancers.

## Supporting Information

S1 Fig Validation of the cell type origin of non-hematopoietic samples. Gene expression for cell type-specific genes as determined by microarray gene expression is shown. PTEC-specific expression is shown for *PAX8*, *KCNIP1*, *KCNJ16* and *FXYD2*; HUVEC-specific expression is shown for *VWF*, *CDH5*, *ESM1* and *CLEC14A*; small intestine-specific expression is shown for *OLFM4*, *RBP2*, *FABP6* and *FAM3B*. Gut-associated expression as defined by detectable expression in gut (both colon and small intestine) as well as a limited number of other non-hematopoietic cell types is shown for *TFF3*, *LGALS4*, *CDH17* and *TACSTD1* and lung-associated expression is demonstrated for *PLUNC*, *SCGB3A1*, *SLPI* and *MSMB*. Probe fluorescence as measured by microarray gene expression analysis is indicated on the x-axis in logarithmic scale.
(PDF)

S2 Fig Clustering analysis of hematopoietic and non-hematopoietic cell types. Hierarchical clustering analysis was performed on all healthy hematopoietic and non-hematopoietic cell types as included in the dataset based on microarray expression profiling of all genes. Hematopoietic cell types were accurately distinguished from non-hematopoietic cell types.
(PDF)

S3 Fig Gene expression for differentiation markers on cell populations isolated from malignant hematopoietic samples. Gene expression for differentiation markers on cell populations isolated from AML, CML, ALL, CLL and MM samples is shown. Black symbols indicate gene expression for surface markers that were used for isolation of malignant cell populations, while grey symbols indicate gene expression for other differentiation markers on isolated cell populations. From AML samples, cell populations were isolated by surface expression of CD33 only (filled circles) or by CD33 in combination with CD14 (open triangles and open squares represent CD33 positive cell populations that are positive or negative for CD14, respectively). Probe fluorescence is indicated on the x-axis in logarithmic scale.
(PDF)

S4 Fig Correlation plots for q-PCR and microarray data. Plots depicting regression models are given for each gene separately. q-PCR values are given on the x-axis and microarray probe fluorescence on the y-axis. For the purpose of graphical representation Cp values were normalized according to reference genes prior to fitting the regression model. Individual R^2^ values derived from the model are depicted in the left upper corner of each plot. Genes are shown in order of maximum probe fluorescence as measured in any cell type of the dataset as selected for q-PCR validation starting with genes with highest maximum probe fluorescence. Each dot represents the mean corrected q-PCR value of duplicate measurements and the probe fluorescence intensity as measured by microarray gene expression analysis. Q-PCR measurements were corrected for expression of reference genes (*HMBS*, *ACTB* and *GAPDH)* in the corresponding sample.
(PDF)

S1 Methods Supplemental methods. (PDF)

S1 Table Complex karyotypes of leukemic cells selected for microarray gene expression analysis. (PDF)

S2 Table Assays and probe IDs for genes included for validation by q-PCR. (PDF)

S3 Table RNA samples used for validation by q-PCR. (PDF)

S4 Table Effect of IFN-γ on gene expression in non-hematopoietic cell types. (PDF)
