# Supplementary figures and images for: Microarray Gene Expression Analysis to Evaluate Cell Type Specific Expression of Targets Relevant for Immunotherapy of Hematological Malignancies
Source: PLoS One. 2016 May 12;11(5):e0155165. doi: 10.1371/journal.pone.0155165 (PMC4865094; doi:10.1371/journal.pone.0155165)

S1 Fig

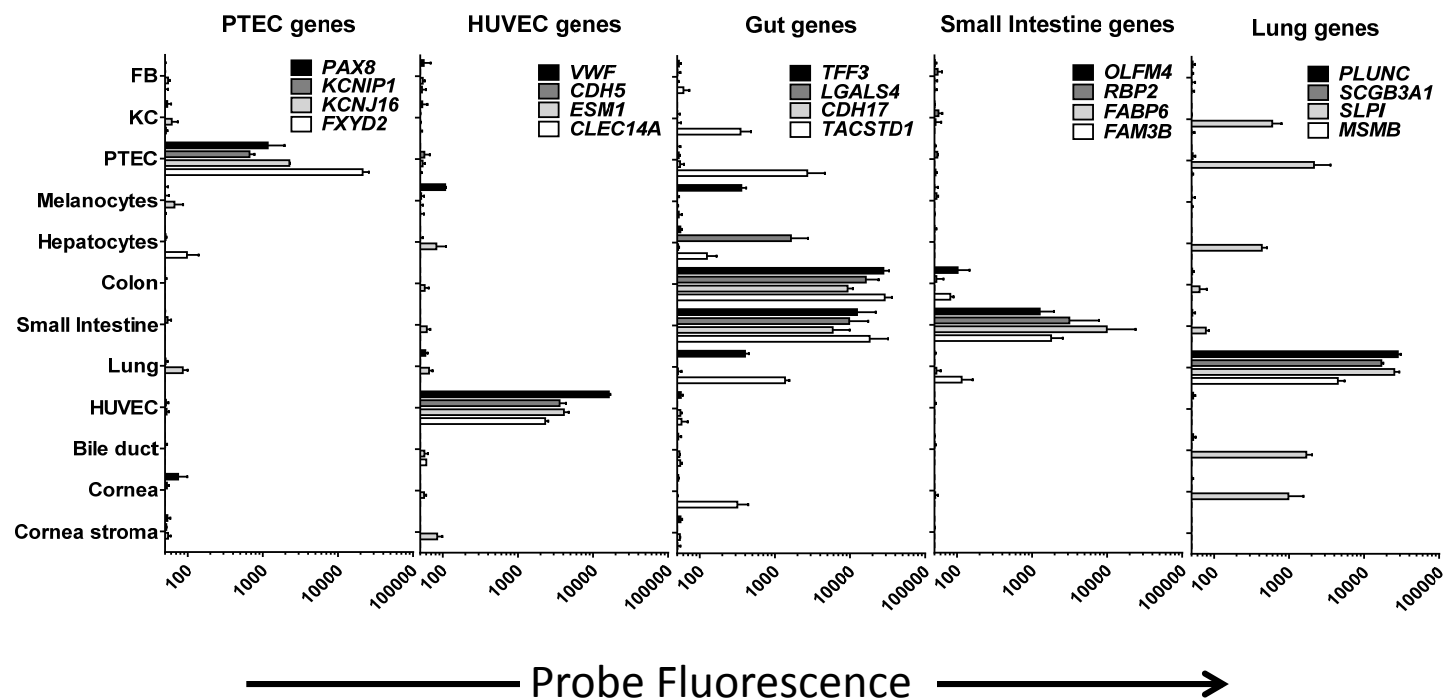

Supplement: S1 Fig — Gene expression for cell type-specific genes as determined by microarray gene expression is shown. PTEC-specific expression is shown for PAX8, KCNIP1, KCNJ16 and FXYD2; HUVEC-specific expression is shown for VWF, CDH5, ESM1 and CLEC14A; small intestine-specific expression is shown for OLFM4, RBP2, FABP6 and FAM3B. Gut-associated expression as defined by detectable expression in gut (both colon and small intestine) as well as a limited number of other non-hematopoietic cell types is shown for TFF3, LGALS4, CDH17 and TACSTD1 and lung-associated expression is demonstrated for PLUNC, SCGB3A1, SLPI and MSMB. Probe fluorescence as measured by microarray gene expression analysis is indicated on the x-axis in logarithmic scale. (PDF) [file pone.0155165.s001.pdf]

# S2 Fig

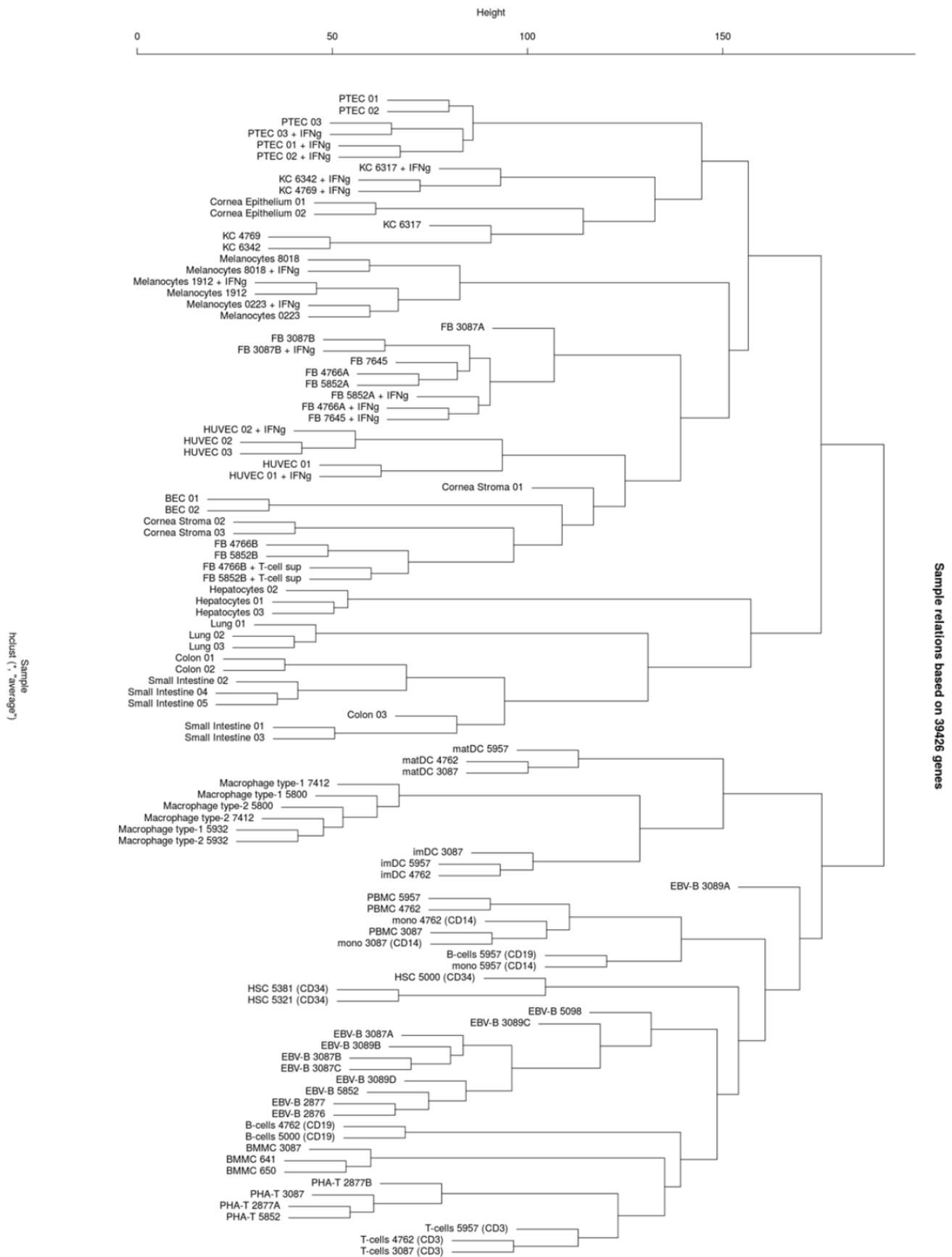

Supplement: S2 Fig — Hierarchical clustering analysis was performed on all healthy hematopoietic and non-hematopoietic cell types as included in the dataset based on microarray expression profiling of all genes. Hematopoietic cell types were accurately distinguished from non-hematopoietic cell types. (PDF) [file pone.0155165.s002.pdf]

S3 Fig

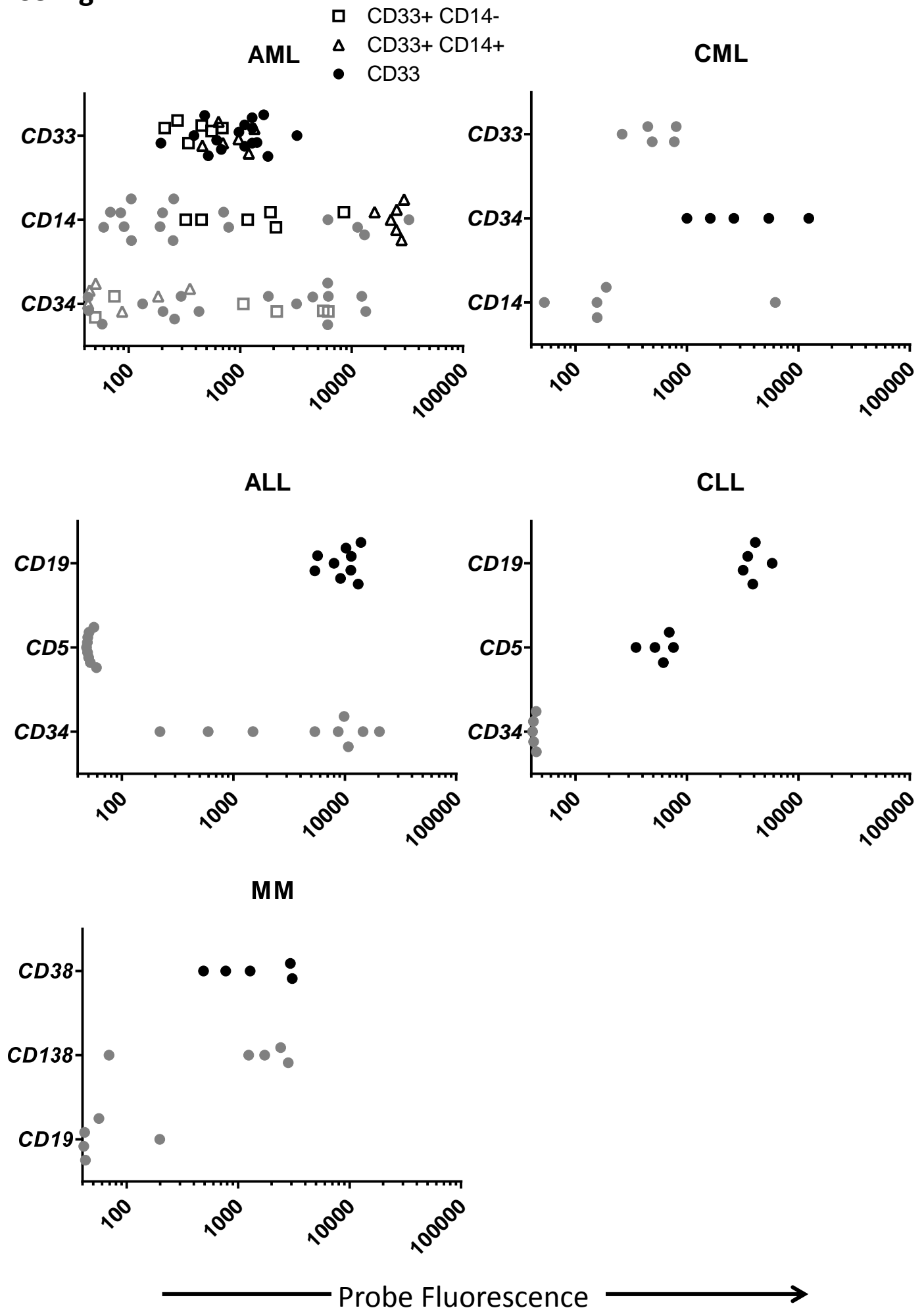

Supplement: S3 Fig — Gene expression for differentiation markers on cell populations isolated from AML, CML, ALL, CLL and MM samples is shown. Black symbols indicate gene expression for surface markers that were used for isolation of malignant cell populations, while grey symbols indicate gene expression for other differentiation markers on isolated cell populations. From AML samples, cell populations were isolated by surface expression of CD33 only (filled circles) or by CD33 in combination with CD14 (open triangles and open squares represent CD33 positive cell populations that are positive or negative for CD14, respectively). Probe fluorescence is indicated on the x-axis in logarithmic scale. (PDF) [file pone.0155165.s003.pdf]

S4 Fig

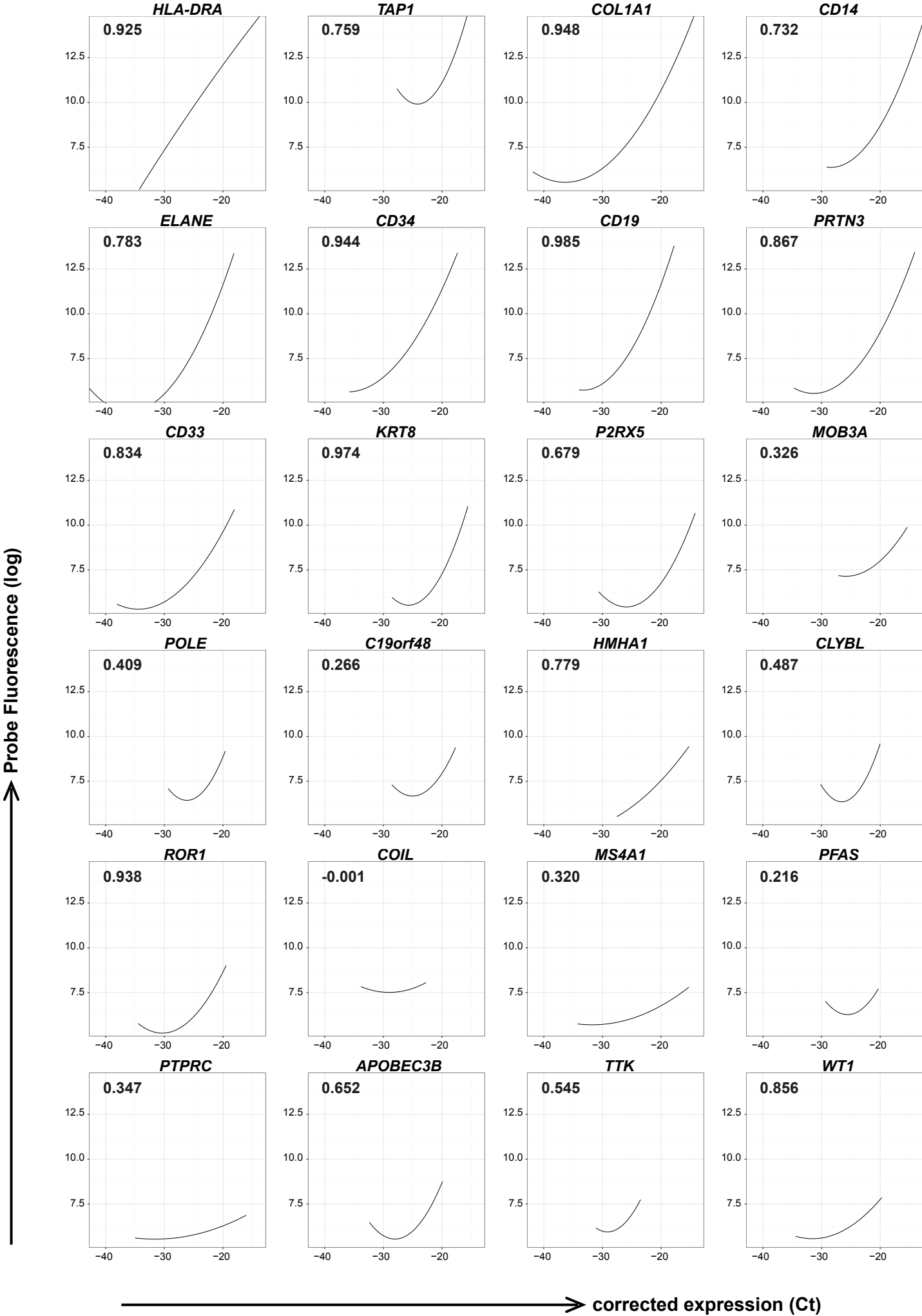

Supplement: S4 Fig — Plots depicting regression models are given for each gene separately. q-PCR values are given on the x-axis and microarray probe fluorescence on the y-axis. For the purpose of graphical representation Cp values were normalized according to reference genes prior to fitting the regression model. Individual R2 values derived from the model are depicted in the left upper corner of each plot. Genes are shown in order of maximum probe fluorescence as measured in any cell type of the dataset as selected for q-PCR validation starting with genes with highest maximum probe fluorescence. Each dot represents the mean corrected q-PCR value of duplicate measurements and the probe fluorescence intensity as measured by microarray gene expression analysis. Q-PCR measurements were corrected for expression of reference genes (HMBS, ACTB and GAPDH) in the corresponding sample. (PDF) [file pone.0155165.s004.pdf]
