# Supplementary material for: Microarray Gene Expression Analysis to Evaluate Cell Type Specific Expression of Targets Relevant for Immunotherapy of Hematological Malignancies
Source: PLoS One. 2016 May 12;11(5):e0155165. doi: 10.1371/journal.pone.0155165 (PMC4865094; doi:10.1371/journal.pone.0155165)
Supplement: S1 Methods — (PDF) [file pone.0155165.s005.pdf]

S1 Methods

Isolation and culture of healthy hematopoietic cells

Bone marrow and peripheral blood mononuclear cells (BMMC and PBMC) were isolated by Ficoll-Isopaque separation and cryopreserved. B-cells, T-cells and monocytes were purified from PBMC by fluorescence-activated cell sorting on a FACS-ARIAIII (BD Biosciences) after staining with monoclonal antibodies (BD Biosciences) for CD19, CD3 and CD14, respectively. Hematopoietic stem cells (HSC) were isolated from G-CSF mobilized peripheral blood by flow cytometric sorting for CD34 surface expression. Immature DC (imDC) were generated by culturing isolated monocytes in medium with 10% human serum (HS) supplemented with 100 ng/mL GM-CSF (Novartis) and 500 IU/mL IL-4 (Schering-Plough) for 5 days. Mature DC (matDC) were generated by culturing imDC for 2 days with 100 ng/mL GM-CSF, 10 ng/mL TNF-α (Cellgenix), 10 ng/mL IL-1β (Cellgenix), 10 ng/mL IL-6 (Cellgenix), 1 μg/mL prostaglandin E2 (Sigma-Aldrich) and 500 IU/mL IFN-γ (Boehringer-Ingelheim). Isolated monocytes were also incubated for 7 days in medium with 10% HS supplemented with 5 ng/ml GM-CSF (Novartis) or 5 ng/ml M-CSF (Chiron) to culture M1 and M2 macrophages (MΦ), respectively. EBV-transformed B-cell lines (EBV-LCL) and PHA-stimulated T-cell lines (PHA-T) were generated from PBMC as previously described[1, 2].

Isolation and culture of malignant hematopoietic cells

Flow cytometric analyses for malignant cells were performed on a FACS-Calibur and cell sorting on a FACS-AriaIII after staining with monoclonal antibodies (all BD Biosciences). Acute lymphoblastic leukemia (ALL) cells were isolated from samples with >85% leukemic blasts (range 85-98%) using antibodies for CD19. Chronic lymphoblastic leukemia (CLL) cells were purified from samples with >90% malignant cells using antibodies for CD19 and CD5. Chronic myeloid leukemia (CML) cells were isolated using antibodies for CD34 from samples with >5% CD34-positive cells. Multiple myeloma cells were sorted from samples with >30% plasma cells (range 30-95%) using antibodies for CD38. Acute myeloid leukemia

(AML) cells were sorted from samples with >20% leukemic blasts (range 22%-95%) as single cell populations or as two separate cell populations expressing CD33 in the absence or presence of CD14 (CD14neg or CD14pos, respectively).

Isolation and culture of non-hematopoietic cells

Fibroblasts and keratinocytes were cultured from skin-biopsies as previously described[1]. PTEC were provided by C. van Kooten (Dept. of Nephrology, LUMC) after isolation from pre-transplant biopsies or from kidneys not suitable for transplantation and culturing in serum-free DMEM/HAM-F12 (Bio-Whittaker, Walkersville, MD) supplemented with antibiotics and insulin, transferrin, selenium, triiodothyronine, epidermal growth factor and hydrocortisone as described earlier[3]. Skin melanocytes were provided by C. Out (Dept. of Dermatology, LUMC) and cultured in F10 supplemented medium (Invitrogen) from surgically removed nevi obtained after informed consent from patients with the atypical nevus syndrome or healthy individuals[4]. PBEC were provided by P.S. Hiemstra (Dept. of Pulmonology, LUMC) and were isolated from macroscopically normal, resected lung tissue from anonymized patients undergoing surgery for lung cancer. Cells were expanded by culture under submerged conditions, and mucociliary differentiation was achieved by culture at the air-liquid interface[5]. HUVEC were provided by H.C. de Boer (Dept. of Nephrology, LUMC) and were isolated from umbilical cords according to Jaffe et al.[6] using a cannula sized to fit the vein. HUVEC were cultured in EGM-2 medium supplemented with the EGM-2 bullet kit (Lonza BioWhittaker, Basel, Switzerland) and refreshed every 3 days.

Cornea stroma and epithelial cells were provided by M.J. Jager (Dept. of Ophthalmology, LUMC) and cultured as previously described[7]. In short, corneal epithelial cells were harvested by cutting the cornea into four equal quadrants, washing the corneal pieces three times in PBS, and incubating the tissue overnight at 4°C in dispase II (Roche Applied Science, Mannheim, Germany). The corneal epithelium was manually separated as a sheet from the underlying tissue, centrifuged, and incubated in trypsin (TrypLE Select, Life Technologies Europe BV, Bleiswijk, the Netherlands) for 10-15 minutes. Single cell

suspensions were cultured in CnT-20 medium (Bio-connect BV, Huissen, the Netherlands) with 1% penicillin/streptomycin. The cornea was cut into small parts of about 1 x 1 mm to isolate cornea stromal cells. The corneal parts were placed in a 0.1% collagenase type II solution (Life Technologies Europe BV) and incubated overnight at 37°C. The obtained cell solution was cultured in DMEM/HAM F12 medium with stable glutamin (Biochrom AG, Berlin, Germany), supplemented with 5% fetal calf serum and 1% penicillin/streptomycin.

Hepatocytes were provided by E. Schmelzer (McGowan Institute for Regenerative Medicine, University of Pittsburgh, USA). Hepatocytes were plated on collagen I-coated culture plates in William's Medium E with glutamax, antibiotics, insulin, transferrin, selenium and hydrocortisone. Medium was renewed daily and hepatocytes were collected at day 2 after two washes with PBS. Colon and small intestinal epithelial cells were provided by R.G. Vries and H. Clevers (Hubrecht Institute for Developmental Biology and Stem Cell Research, University Medical Centre Utrecht, Utrecht, The Netherlands) and cultured as described previously[8]. Bile duct epithelial cells (SC-5100, passage 4) were purchased from ScienCell (Carlsbad, CA, USA) and cultured according to manufacturer's instructions.

Fibroblasts, keratinocytes, PTEC, melanocytes and HUVEC were also cultured in the presence of IFN-γ (100 IU/ml) for 4 days to mimic an inflammatory environment. Additionally, two fibroblast samples were cultured with a T-cell culture supernatant for 4 days. This supernatant was collected from a CD4 T cell clone that was stimulated with leukemic-antigen presenting cells[9] and contained high levels of IFN-γ (3100 pg/ml), IL-13 (500 pg/ml), TNF-α (700 pg/ml) and IL-2 (600 pg/ml) as measured by multi-cytokine ELISA.

References

1. Griffioen M, Honders MW, van der Meijden ED, van Luxemburg-Heijs SA, Lurvink EG, Kester MG, et al. Identification of 4 novel HLA-B*40:01 restricted minor histocompatibility antigens and their potential as targets for graft-versus-leukemia reactivity. Haematologica. 2012;97(8):1196-204.
2. Stumpf AN, van der Meijden ED, van Bergen CA, Willemze R, Falkenburg JH, Griffioen M. Identification of 4 new HLA-DR-restricted minor histocompatibility antigens as hematopoietic targets in antitumor immunity. Blood. 2009;114(17):3684-92.
3. van Kooten C, Gerritsma JS, Paape ME, van Es LA, Banchereau J, Daha MR. Possible role for CD40-CD40L in the regulation of interstitial infiltration in the kidney. Kidney Int. 1997;51(3):711-21.
4. Gao L, van Nieuwpoort FA, Out-Luiting JJ, Hensbergen PJ, de Snoo FA, Bergman W, et al. Genome-Wide Analysis of Gene and Protein Expression of Dysplastic Naevus Cells. Journal of Skin Cancer. 2012;2012:13.
5. Amatngalim GD, van Wijck Y, de Mooij-Eijk Y, Verhoosel RM, Harder J, Lekkerkerker AN, et al. Basal cells contribute to innate immunity of the airway epithelium through production of the antimicrobial protein RNase 7. J Immunol. 2015;194(7):3340-50.
6. Jaffe EA, Nachman RL, Becker CG, Minick CR. Culture of human endothelial cells derived from umbilical veins. Identification by morphologic and immunologic criteria. J Clin Invest. 1973;52(11):2745-56.
7. van Essen TH, van Zijl L, Possemiers T, Mulder AA, Zwart SJ, Chou CH, et al. Biocompatibility of a fish scale-derived artificial cornea: Cytotoxicity, cellular adhesion and phenotype, and in vivo immunogenicity. Biomaterials. 2016;81:36-45.
8. Sato T, Stange DE, Ferrante M, Vries RG, Van Es JH, Van den Brink S, et al. Long-term expansion of epithelial organoids from human colon, adenoma, adenocarcinoma, and Barrett's epithelium. Gastroenterology. 2011;141(5):1762-72.
9. Stevanovic S, Nijmeijer BA, van Schie ML, Salvatori DC, Maas S, Griffioen M, et al. Donor T Cells Administered Over HLA Class II Barriers Mediate Antitumor Immunity without Broad Off-Target Toxicity in a NOD/Scid Mouse Model of Acute Leukemia. Biol Blood Marrow Transplant. 2013;19(6):867-75.
