# Supplementary material for: Microarray Gene Expression Analysis to Evaluate Cell Type Specific Expression of Targets Relevant for Immunotherapy of Hematological Malignancies
Source: PLoS One. 2016 May 12;11(5):e0155165. doi: 10.1371/journal.pone.0155165 (PMC4865094; doi:10.1371/journal.pone.0155165)
Supplement: S1 Table — (PDF) [file pone.0155165.s006.pdf]

S1 Table: Complex karyotypes of leukemic cells selected for microarray gene expression analysis

Sample[a]	Complex karyotype[b]
AML 4781 (CD33/CD14)	46,XY,inv(3)(q21q26),del(7)(q21),-17,der(19)t(17;19)(q2?2;q13)[cp6]/ 46~47,XY,del(2)(p21),-3,del(3)(q23),+6,-7,del(11)(p1?3),add(12)(p11),inv(15)(q?13q?23),+ring,+mar[cp11]/ 46,XY[4]
AML 3714 (CD33)	45-46, XX, ?inv (3) (p?q?), -4, add (5) (q1?2), der (9) t(4;9) (q21;q34), ?dic (11;?) (p1?2;?), add (12) (p13), der (12) add (12) (p?) del (12) (q22), add (14) (q13), -17, del (17) (q21), +1-2mar [19] / 46, XX [1]
AML 3778 (CD33)	47, XX, +8, del (12) (p13) [5] / 47, idem, i (17) (q10) [7] / 47, XX, +add (8) (q24), del (12) (p13), i (17) (q10) [2]
AML 5074 (CD33/CD14)	47, XY, +3 [3] / 48, idem, +20 [2] / 46, XY, t(10;16) (q21;p13) [3]
ALL 5903 (CD19)	46, XX, t(9;22) (q34;q11) [6] / 45, XX, -7, t(9;22) (q34;q11), add (14) (p11) [2] / 46, XX [7]
ALL 3655 (CD19)	85-88, XX, +1, -3, -4, -5, -6, -7, -7, -8, -9, -9, -10, +11, +12, -13, -14, -15, -15, -16, -17, -17, -18. +6-14mar, inc [cp16] / 46, XX [1]
CML 3471 (CD34)	46, XY, t(9;22) (q34;q11) .ish der(9) t(9;22) del(9) (q34q34) (ABL-,WCP22+), der(22) t(9;22) (BCR+, ABL+, WCP22+) [19] /46, XY [1]
CML 3087 (CD34)	46, XX, t(9;22) (q34;q11) [10]/46, X, del(X) (p11.?4), t(9;22) (q34;q11) [13]/46, X, del(X) (p11.?4), t(9;22) (q34;q11), del(17) (q11.2q21) [5]/46, XX [1]
CLL 4725 (CD19/CD5)	45, XY, -10, ?der (17;22) (q10;q10), +mar [5] / 45, XY, -10, ?der (17;18) (q10;q10), +mar [cp3] / 46, XY [2] / 44-46, XY, +1-2 mar [cp5]]
MM 5987 (CD38)	46-47, X, -Y, del(1) (p13), +6, del(6) (q1?6), del(8) (p1?2), -11, -12, -12, -13, add(14) (p11), +2-4mar [cp2]
MM 5744 (CD38)	43, X -X, der(2) t(1;2) (q11;q37), -10, -13, -14, +mar [2] / 46,XX [7]
MM 5019 (CD38)	40-43, XY, del (1) (p13), add (6) (q21), add (8) (q24), add (9) (q34), add (12) (p11), -13, der (14) t(11;14) (q13;q32), -16, add (18) (q23), der (19) del (19) (p13) del (19) (q13), -21, -22 [cp16] / 46, XY [4]

[a] Markers for flow cytometric isolation of malignant cells are indicated between brackets. AML cells have been isolated by expression of CD33 (CD33) or a combination of CD33 and CD14 (CD33/CD14) in which CD33 expressing AML cells positive or negative for monocyte lineage differentiation marker CD14 have been selected as two separate cell populations. CLL cells have been selected for co-expression of CD19 and CD5. ALL, CML and MM cells have been isolated by expression of CD19, CD34 and CD38, respectively.

[b] Complex karyotypes are depicted for unstimulated and/or stimulated cells.
