# Supplementary material for: Microarray Gene Expression Analysis to Evaluate Cell Type Specific Expression of Targets Relevant for Immunotherapy of Hematological Malignancies
Source: PLoS One. 2016 May 12;11(5):e0155165. doi: 10.1371/journal.pone.0155165 (PMC4865094; doi:10.1371/journal.pone.0155165)
Supplement: S2 Table — (PDF) [file pone.0155165.s007.pdf]

S2 Table: Assays and probe IDs for genes included for validation by q-PCR

Set[a]	Genes[b]	Taqman assay[c]	Probe 1[d]	Probe 2	Probe 3
1	PRTN3	Hs01597752_m1	ILMN_1753584		
1	ELANE	Hs00975994_g1	ILMN_1706635		
1	CD33	Hs01076281_m1	ILMN_1747622		
1	CD34	Hs00990732_m1	ILMN_1732799	ILMN_2341229	
1	TAP1	Hs00388675_m1	ILMN_1751079		
1	CD14	Hs02621496_s1	ILMN_1740015	ILMN_2396444	
1	HLA-DRA	Hs00219575_m1	ILMN_1689655	ILMN_2157441	
1	PTPRC	Hs04189704_m1	ILMN_2340217	ILMN_1653652	
1	HMHA1	Hs00943375_m1	ILMN_1811392		
1	COL1A1	Hs00164004_m1	ILMN_1701308		
1	CD19	Hs01047410_g1	ILMN_1782704		
1	KRT8	Hs02339474_g1	ILMN_1753584		
2	APOBEC3B	Hs00358981_m1	ILMN_2219466		
2	C19orf48	Hs01066105_m1	ILMN_1759184	ILMN_2383484	
2	CLYBL	Hs00370518_m1	ILMN_1663538		
2	COIL	Hs00982300_m1	ILMN_1688034		
2	MOB3A	Hs00926925_m1	ILMN_1721344		
2	MS4A1	Hs00544819_m1	ILMN_1776939		
2	P2RX5	Hs01112471_m1	ILMN_1677793		
2	PFAS	Hs00389822_m1	ILMN_1755862		
2	POLE	Hs00173030_m1	ILMN_1728199		
2	ROR1	Hs00938677_m1	ILMN_1655904		
2	TTK	Hs01009870_m1	ILMN_1788166		
2	WT1	Hs01103751_m1	ILMN_1802174		
ref[e]	HMBS	Hs00609293_g1	ILMN_1685954	ILMN_1726306	
ref[e]	ACTB	Hs99999903_m1	ILMN_1777296	ILMN_2038777	ILMN_2152131
ref[e]	GAPDH	Hs99999905_m1	ILMN_1343295	ILMN_1802252	ILMN_2038778

[a] Set indicates in which q-PCR validation sample set the gene was included.

[b] Official gene symbols are depicted for genes included in the q-PCR validation set.

[c] Predesigned Taqman gene expression assays were obtained from Thermo Fisher Scientific.

[d] Illumina probe IDs as included in the microarray database.

[e] Ref indicates genes that are used as reference genes for q-PCR.
