# Supplementary material for: Microarray Gene Expression Analysis to Evaluate Cell Type Specific Expression of Targets Relevant for Immunotherapy of Hematological Malignancies
Source: PLoS One. 2016 May 12;11(5):e0155165. doi: 10.1371/journal.pone.0155165 (PMC4865094; doi:10.1371/journal.pone.0155165)
Supplement: S3 Table — (PDF) [file pone.0155165.s008.pdf]

S3 Table: RNA samples used for validation by q-PCR

Number	RNA samples[a]	Used in set[b]
1	ALL 2375 (CD19)	1 & 2
2	ALL 2391 (CD19)	1
3	ALL 1833 (CD19)	1 & 2
4	ALL 1299 (CD19)	2
5	ALL 3281 (CD19)	2
6	AML 3714 (CD33)	1 & 2
7	AML 2467 (CD33/CD14pos)	1
8	AML 1310 (CD33/CD14pos)	1
9	AML 1310 (CD33/CD14neg)	1 & 2
10	CML 2195 (CD34)	1
11	CML 5036 (CD34)	1
12	CLL 4725 (CD19/CD5)	2
13	CLL 2159 (CD19/CD5)	2
14	MM 5987 (CD38)	2
15	MM 5744 (CD38)	2
16	PBMC 5957	1
17	PBMC 4762	1
18	B-cells 5957 (CD19)	1 & 2
19	B-cells 4762 (CD19)	1 & 2
20	T-cells 3087 (CD3)	1
21	HSC 5381 (CD34)	1 & 2
22	HSC 5321 (CD34)	1 & 2
23	Mono 5957 (CD14)	1 & 2
24	Mono 4762 (CD14)	1
25	Mono 3087 (CD14)	2
26	matDC 5957	1 & 2
27	matDC 4762	1
28	matDC 3087	2
29	Cornea Stroma 02	1
30	Cornea Stroma 03	1
31	BEC 01	1
32	BEC 02	1
33	FB 5852A	1 & 2
34	FB 7645	1
35	FB 3087B	2
36	FB 5852A + IFN-γ	1 & 2
37	FB 7645 + IFN-γ	1
38	FB 3087B + IFN-γ	2
39	KC 6317	1 & 2
40	KC 6342	1 & 2
41	KC 6317 + IFN-γ	1
42	KC 6342 + IFN-γ	1
43	EBV-B 3089A	2
44	EBV-B 3089C	2
45	EBV-B 2876	2
46	EBV-B 5852	2
47	EBV-B 3087A	2
48	Colon 02	2
49	Colon 03	2
50	BEC 01	2
51	BEC 02	2
52	Hepatocytes 01	2
53	Hepatocytes 02	2

[a] RNA samples for q-PCR were the same as used for microarray gene expression analysis and were stored at -80°C. [b] Used in set indicates in which q-PCR validation set the samples were included.
