# Supplementary material for: Microarray Gene Expression Analysis to Evaluate Cell Type Specific Expression of Targets Relevant for Immunotherapy of Hematological Malignancies
Source: PLoS One. 2016 May 12;11(5):e0155165. doi: 10.1371/journal.pone.0155165 (PMC4865094; doi:10.1371/journal.pone.0155165)
Supplement: S4 Table — (PDF) [file pone.0155165.s009.pdf]

S4 Table: Gene expression as induced in different non-hematopoietic cells by IFN-γ

Probe ID[a]	Gene[b]	FB[c]		FB + IFN-γ[d]		KC[e]		KC + IFN-γ[f]		PTEC[g]		PTEC + IFN-γ[h]		MEL[i]		MEL + IFN-γ[j]		HUVEC[k]		HUVEC + IFN-γ[l]	
		AVG[m]	SD[m]	AVG[n]	SD	AVG	SD	AVG	SD	AVG	SD	AVG	SD	AVG	SD	AVG	SD	AVG	SD	AVG	SD
ILMN_2329927	ABCG1	48.84	0.1	49.90	5.8	81.75	21.1	**1035.69**	252.9	50.06	1.6	54.51	6.9	44.59	2.0	48.09	4.9	150.05	21.7	98.93	16.9
ILMN_1763666	ALDH3B2	49.61	0.1	46.71	2.6	189.23	44.3	**5453.54**	1219.8	46.83	3.6	50.84	4.3	43.76	4.3	48.32	4.6	38.85	0.2	41.77	0.5
ILMN_2363880	ALDH3B2	54.16	0.2	52.95	2.7	72.33	11.1	**962.44**	427.3	54.65	2.4	55.87	2.2	56.12	5.5	58.23	4.1	45.37	4.2	41.72	3.1
ILMN_1692332	ALOX12B	51.75	0.1	51.18	3.6	54.84	5.8	**933.92**	134.3	52.97	2.3	51.53	4.0	48.58	2.3	48.44	0.9	45.34	1.1	40.87	4.1
ILMN_1756862	APOL3	159.03	0.6	**1947.70**	459.5	49.63	6.4	**640.93**	208.9	75.73	10.1	404.24	81.3	45.81	1.2	59.00	14.6	194.68	160.5	1638.69	590.1
ILMN_1762284	ASPRV1	58.08	0.0	57.51	5.0	53.05	2.2	**1152.97**	459.9	55.18	4.3	60.09	5.6	72.56	7.9	68.28	5.4	49.69	1.0	49.94	1.1
ILMN_1723480	BST2	115.21	1.0	602.93	306.3	67.15	14.4	**898.60**	138.7	702.79	229.8	1569.08	273.2	227.37	96.1	384.41	129.3	511.90	506.5	1248.34	1181.2
ILMN_1677198	C1R	1852.86	1.2	4264.54	2880.8	156.71	67.2	1299.28	248.7	264.47	256.3	1632.03	596.7	56.32	12.4	101.82	30.5	52.36	5.5	**987.57**	259.9
ILMN_1764109	C1R	1709.28	1.4	4510.50	3015.5	111.55	66.5	**1211.92**	247.7	232.46	200.7	1218.73	332.8	62.81	6.4	101.00	32.1	58.07	1.2	**733.80**	113.5
ILMN_1781626	C1S	2733.16	0.6	10171.05	3994.1	113.80	22.5	846.83	313.2	225.07	179.0	**2447.75**	1696.2	54.04	4.2	86.21	17.2	63.89	3.8	**1524.57**	649.4
ILMN_1773352	CCL5	54.39	0.2	73.26	20.2	54.55	1.3	**2737.69**	1472.4	60.95	11.7	182.60	56.6	91.06	16.9	106.04	69.7	60.86	0.4	58.66	2.9
ILMN_2098126	CCL5	57.79	0.2	72.36	18.0	61.30	8.7	**1980.55**	706.8	73.48	26.5	263.78	109.3	114.04	35.4	150.66	83.7	88.92	3.4	82.33	5.3
ILMN_1736567	CD74	77.09	0.8	**3767.31**	1778.8	68.31	25.1	**7122.15**	3193.4	301.65	91.9	**15682.34**	3869.4	58.57	2.8	**1633.06**	845.3	50.41	3.5	**6673.13**	1222.4
ILMN_2379644	CD74	61.66	0.6	**1642.13**	898.2	53.63	3.4	**2318.52**	842.6	133.58	56.4	**4314.29**	716.8	44.68	5.7	**560.01**	220.9	44.12	1.2	**2848.28**	1142.9
ILMN_1761464	CD74	47.12	0.2	**985.43**	776.3	49.35	6.0	**2704.66**	1553.0	69.99	14.4	**1939.27**	899.9	44.81	7.4	249.88	77.0	40.52	2.3	309.32	151.4
ILMN_1712522	CEACAM6	45.48	0.1	43.42	4.6	48.79	7.1	**985.71**	977.1	67.23	29.2	70.09	36.1	41.14	3.3	43.02	4.0	46.19	1.3	44.51	2.8
ILMN_1774287	CFB	304.78	0.9	1014.58	450.3	128.66	14.3	**5047.12**	1322.1	189.58	66.8	1658.66	530.1	54.19	4.3	61.49	7.3	83.75	22.2	353.69	164.0
ILMN_1803838	CNFN	93.28	0.3	75.92	21.3	196.52	45.6	**8871.86**	1546.6	81.94	2.7	66.44	2.5	109.86	12.3	92.76	20.6	72.91	13.3	64.48	14.4
ILMN_1803452	CRCT1	61.52	0.2	55.13	2.0	119.52	57.9	**4816.55**	2047.1	47.39	0.7	51.37	2.4	50.83	6.0	50.52	6.5	55.02	0.4	54.00	4.0
ILMN_1697733	CST6	103.71	1.2	53.76	10.2	99.92	46.5	**2001.80**	1302.7	143.03	58.2	174.10	100.7	49.59	2.9	48.88	4.0	55.37	2.6	55.20	6.3
ILMN_1698666	CST6	79.79	0.8	50.15	3.7	127.88	74.4	**1807.84**	1407.5	138.67	40.1	202.76	100.5	45.67	1.9	44.24	4.7	44.30	0.3	45.73	1.2
ILMN_1654072	CX3CL1	49.39	0.1	56.89	4.3	134.20	47.6	**2110.01**	827.4	403.92	166.7	2121.91	962.7	51.93	3.4	50.39	1.7	133.02	43.4	649.37	600.8
ILMN_1791759	CXCL10	50.13	0.1	187.09	128.1	136.35	96.2	**17124.49**	8744.2	54.74	10.1	**2037.19**	1558.5	53.98	10.1	87.52	21.7	69.02	0.6	**15285.57**	16284.2
ILMN_1745356	CXCL9	54.28	0.2	214.43	158.3	41.93	6.0	**4979.47**	4668.8	48.87	4.8	**507.69**	571.4	50.43	2.1	89.30	29.9	44.66	1.0	**14988.97**	19786.6
ILMN_1686573	DEFB1	51.22	0.1	52.32	5.9	633.89	236.4	**10526.60**	6622.4	1816.05	1946.7	883.34	565.2	45.94	8.1	45.16	5.3	47.79	2.7	53.22	1.9
ILMN_1684308	DEFB103B	55.06	0.2	53.17	4.1	63.96	13.8	**7264.68**	7549.3	49.21	2.3	54.45	4.7	52.69	4.5	49.26	2.8	58.58	0.7	52.89	1.0
ILMN_1714587	DEFB103B	50.71	0.1	49.86	2.7	53.23	4.7	**2182.45**	1939.4	46.29	2.5	50.05	2.4	45.53	1.2	46.63	2.2	46.71	2.9	47.94	3.2
ILMN_1733998	DHRS9	52.63	0.1	53.11	9.8	79.20	10.4	**2211.26**	2488.2	78.67	25.3	54.43	7.6	49.61	2.9	50.49	2.9	64.24	1.4	74.75	2.5
ILMN_1769201	ELF3	47.54	0.1	49.41	4.0	63.21	9.5	**739.08**	506.2	867.37	122.3	1078.05	154.1	56.94	6.1	56.35	0.9	56.94	2.6	61.41	1.1
ILMN_1671600	EPS8L1	49.09	0.2	46.40	4.6	83.61	24.7	**879.20**	406.4	95.58	29.4	82.11	24.3	114.34	29.4	88.67	16.7	104.74	63.1	93.78	52.4
ILMN_2388547	EPSTI1	113.74	0.7	**1647.95**	506.8	317.50	175.0	2245.49	275.1	350.78	64.1	3297.06	297.7	366.54	214.8	498.67	112.1	85.62	20.6	**993.58**	415.0
ILMN_1701114	GBP1	173.11	0.4	**2023.51**	706.8	150.79	84.2	1135.13	297.1	200.54	44.8	**2493.85**	1102.2	128.84	95.6	827.20	273.8	134.26	62.1	**3115.13**	2505.9
ILMN_2148785	GBP1	125.72	0.4	1239.64	132.5	116.18	39.8	497.33	118.5	109.20	44.3	**1258.73**	406.2	78.70	31.8	538.87	248.4	150.13	42.2	**3480.33**	2692.5
ILMN_1774077	GBP2	603.04	0.8	**12958.95**	5995.9	89.61	17.4	**3991.82**	1595.7	1435.90	760.5	**16358.86**	7106.1	135.98	28.5	**1920.36**	953.9	518.73	260.1	**9395.13**	5650.8
ILMN_1771385	GBP4	48.66	0.2	**725.83**	211.4	51.44	4.5	**902.42**	195.9	71.79	15.3	**2237.92**	305.7	50.86	5.2	124.59	67.2	72.91	11.6	**2401.53**	308.5
ILMN_2114568	GBP5	55.38	0.1	**892.66**	507.3	60.87	24.8	**1138.56**	226.2	46.33	3.7	**5276.27**	789.8	44.24	1.1	50.40	6.7	44.09	2.1	**2017.36**	1414.2
ILMN_2188862	GDF15	1059.99	0.4	1072.21	600.2	198.91	159.0	**3104.88**	2204.0	2550.20	2171.0	5469.76	2321.4	726.40	235.3	2450.76	2072.3	4771.94	1280.1	3503.67	216.0
ILMN_1803945	HCP5	77.79	0.4	**958.35**	460.8	656.35	301.3	3072.55	1779.9	254.44	65.1	2029.85	535.5	449.64	451.6	1056.99	905.8	48.92	0.9	**569.00**	235.4
ILMN_1758623	HIST1H2BD	210.09	0.3	334.05	105.0	295.16	103.9	**4056.45**	2697.8	94.68	15.9	109.31	33.9	649.55	153.6	617.30	34.9	61.36	1.3	62.48	9.3
ILMN_1651496	HIST1H2BD	287.15	0.3	420.55	176.9	409.24	193.7	**5452.04**	3516.2	103.39	4.6	132.37	46.8	1063.49	169.2	1013.49	66.1	68.91	5.1	70.99	16.6
ILMN_1778401	HLA-B	907.11	0.9	**9361.50**	1594.6	1635.81	252.0	**17150.88**	6003.9	2165.09	1430.9	14946.59	964.3	2928.13	2434.1	11274.35	2700.4	305.69	119.6	**8580.11**	3072.6
ILMN_1695311	HLA-DMA	396.45	0.8	**6931.34**	2366.2	236.17	111.9	**4290.23**	960.5	296.47	53.2	**4341.77**	2171.5	268.77	72.6	**4124.57**	415.3	118.99	46.0	**2158.77**	2130.0
ILMN_1761733	HLA-DMB	65.93	0.7	**1780.86**	967.1	56.33	12.8	**2044.10**	785.5	1072.82	206.4	3251.26	1203.6	62.33	20.3	**737.00**	40.8	63.16	5.6	**1047.80**	845.0
ILMN_1659075	HLA-DOA	50.75	0.1	**1231.98**	741.6	44.49	8.9	247.60	138.4	46.69	1.5	**852.73**	754.7	46.01	2.5	251.80	72.0	51.47	6.1	376.36	230.0
ILMN_1772218	HLA-DPA1	116.29	0.8	**5347.41**	3408.7	45.00	1.4	**5898.30**	3368.2	67.18	6.3	**3476.02**	2001.4	226.50	192.2	**2819.46**	1753.3	55.11	6.1	**1527.72**	1152.5
ILMN_1808405	HLA-DQA1	55.12	0.2	**1200.68**	1121.2	43.22	2.7	**1594.54**	1138.9	44.10	1.9	**1238.75**	960.5	43.13	6.5	309.54	147.8	41.21	1.4	**455.84**	448.5
ILMN_2157441	HLA-DRA	96.33	1.0	**5896.75**	2407.4	50.73	2.2	**8090.95**	751.2	64.95	14.3	**7896.86**	3547.8	59.93	10.1	**3979.94**	923.2	54.50	1.6	**4817.07**	2652.6
ILMN_1689655	HLA-DRA	120.13	1.2	**13994.52**	4867.1	51.71	3.1	**16149.33**	6775.5	69.65	14.1	**8802.78**	5386.2	55.33	8.7	**4770.78**	1192.5	72.54	6.4	**3582.60**	2716.6
ILMN_1715169	HLA-DRB1	52.87	0.3	272.20	263.5	45.56	5.0	316.79	227.4	53.89	3.2	**638.04**	41.7	47.29	5.5	297.85	212.7	54.96	0.2	57.51	1.1

Probe ID[a]	Gene[b]	FB[c]		FB + IFN-γ[d]		KC[e]		KC + IFN-γ[f]		PTEC[g]		PTEC + IFN-γ[h]		MEL[i]		MEL + IFN-γ[j]		HUVEC[k]		HUVEC + IFN-γ[l]	
		AVG[m]	SD[m]	AVG[n]	SD	AVG	SD	AVG	SD	AVG	SD	AVG	SD	AVG	SD	AVG	SD	AVG	SD	AVG	SD
ILMN_1752592	HLA-DRB4	80.20	0.5	**4722.43**	3240.0	63.89	14.3	**9081.97**	10653.3	61.87	5.8	**2789.86**	483.3	59.24	1.3	**3116.55**	2734.7	62.92	4.3	**2740.59**	2753.1
ILMN_1697499	HLA-DRB5	61.78	0.5	439.61	775.9	48.06	3.4	**1972.46**	2024.2	47.41	3.7	**2414.16**	1278.0	45.96	6.1	**1061.59**	908.5	46.57	2.8	48.44	4.4
ILMN_2066066	HLA-DRB6	52.99	0.1	231.58	66.0	56.97	10.1	396.13	36.8	56.42	1.2	371.08	101.0	55.20	7.1	264.19	36.0	49.87	5.4	**702.18**	568.2
ILMN_2186806	HLA-F	216.76	0.6	815.38	143.3	364.69	75.9	2206.76	533.1	371.17	245.1	2035.55	946.4	519.52	295.8	1016.74	572.3	194.52	72.4	**1946.75**	103.7
ILMN_2130441	HLA-H	1416.17	0.6	9505.64	1554.9	2118.79	133.5	11458.84	1271.5	2174.33	1698.4	13557.52	3087.1	4030.01	3242.4	10037.66	5575.5	651.59	351.7	**9103.70**	171.5
ILMN_2316236	HOPX	45.94	0.1	42.92	5.1	138.08	76.0	**2507.79**	432.2	58.52	3.8	41.71	1.0	48.73	4.7	44.02	3.5	147.44	91.6	226.15	173.8
ILMN_2058782	IFI27	632.45	0.9	**8740.06**	8071.3	14560.92	9119.4	25672.22	808.3	229.73	293.4	**2802.31**	1266.4	8251.50	9949.4	8078.30	4960.9	5575.76	389.9	7839.86	5570.8
ILMN_1723912	IFI44L	88.38	0.7	**1204.84**	453.2	420.66	327.9	1397.99	544.1	254.71	135.9	**3737.78**	296.1	2091.36	981.5	3245.26	692.0	131.47	37.5	**1500.45**	753.1
ILMN_1739428	IFIT2	164.99	0.2	**2214.68**	576.0	212.66	88.9	**2151.56**	1623.0	224.64	112.9	2156.26	265.6	1110.65	480.2	2026.69	486.0	61.63	0.6	176.29	27.0
ILMN_1701789	IFIT3	277.23	0.5	**5719.26**	1860.9	600.17	448.6	5257.17	2311.6	339.85	126.6	**4337.01**	354.1	3155.73	1894.5	6597.25	2651.4	60.53	6.3	**732.62**	220.4
ILMN_1801246	IFITM1	1117.55	1.2	9494.69	3342.8	3503.69	1844.2	17076.49	837.7	4407.33	2726.6	23031.44	893.8	3985.89	3134.6	14673.66	5747.0	155.79	87.8	**1637.90**	1283.6
ILMN_2334296	IL18BP	121.12	0.1	**4541.91**	2733.1	73.80	19.9	**1258.71**	223.4	145.65	38.5	**8542.26**	2796.2	85.65	4.7	541.61	280.5	272.38	157.1	**3289.94**	212.3
ILMN_1804901	IL1F5	49.36	0.2	52.07	7.8	72.91	6.4	**888.23**	86.5	49.05	2.4	54.00	2.2	52.98	4.0	55.31	3.1	42.38	3.4	41.52	2.2
ILMN_2368530	IL32	57.32	0.2	210.39	74.3	74.24	6.8	**1227.70**	225.6	608.33	531.3	1470.46	1547.2	51.85	6.1	52.15	10.6	1922.19	1012.3	2679.37	1611.7
ILMN_1778010	IL32	78.33	0.2	356.41	214.5	79.44	17.2	**1033.18**	452.3	454.47	337.1	815.64	698.4	53.89	3.4	51.30	0.8	526.22	456.5	686.87	601.3
ILMN_1656310	INDO	45.82	0.3	**525.29**	132.5	59.43	15.1	340.77	266.9	59.23	14.7	**1360.05**	819.0	49.24	4.0	86.92	18.1	44.89	0.2	**2549.94**	1035.2
ILMN_1708375	IRF1	378.04	0.8	2621.77	246.6	196.84	19.1	**4635.05**	2186.4	1053.01	505.7	5612.52	2483.5	221.94	84.3	1715.14	649.4	214.73	193.6	**2441.88**	179.7
ILMN_1695924	KLK11	53.19	0.2	50.59	4.5	802.88	179.3	**10005.51**	2019.0	47.30	4.5	50.59	3.4	49.22	4.6	47.91	4.3	55.29	5.9	50.29	3.1
ILMN_1745570	KLK7	55.77	0.2	64.67	8.1	1300.40	728.1	**15106.71**	6679.6	84.20	49.4	68.63	11.9	56.67	3.9	56.57	9.1	48.37	2.7	47.63	1.7
ILMN_1791545	KRT23	74.38	0.1	70.16	13.3	124.39	42.6	**2088.33**	1200.5	85.89	26.6	64.48	6.9	104.50	12.7	88.69	16.1	47.56	1.6	47.43	4.3
ILMN_1705814	KRT80	77.66	0.5	48.93	5.6	87.15	29.0	**7107.21**	2982.1	551.53	267.0	197.97	88.2	50.82	3.3	48.89	2.5	303.35	22.4	223.93	1.7
ILMN_2170814	LAMP3	55.19	0.4	119.83	127.9	76.10	21.9	**1048.95**	275.9	53.25	9.0	**549.14**	145.3	48.29	4.8	64.23	12.4	323.06	26.2	419.63	22.6
ILMN_1808220	LCE3E	66.39	0.3	64.63	14.8	56.19	7.2	**802.66**	396.4	53.53	9.7	65.71	12.2	60.13	6.0	58.36	3.6	51.24	2.6	51.57	4.3
ILMN_1659688	LGALS3BP	544.76	0.8	1396.51	911.7	644.97	299.3	1007.35	384.4	1181.26	513.3	2832.00	660.7	3228.87	1842.7	4620.85	2686.5	45.16	1.2	**480.59**	92.3
ILMN_2412214	LGALS9	67.59	0.4	314.35	196.5	56.63	5.6	56.83	5.9	67.32	6.6	198.68	59.5	87.12	27.7	92.45	39.4	94.55	34.4	**975.77**	235.6
ILMN_2332964	LGMN	627.24	0.5	560.58	70.3	424.37	80.7	**5382.45**	2008.1	505.11	46.6	1106.87	235.8	319.24	47.5	419.66	67.4	470.90	38.3	681.96	106.8
ILMN_1815895	LOC649143	48.16	0.1	**496.09**	411.0	46.77	4.4	**995.65**	242.8	46.62	3.8	**714.91**	792.5	47.59	1.4	**604.77**	769.9	58.81	2.1	**880.51**	810.9
ILMN_1722670	LYNX1	48.19	0.2	46.55	5.1	54.21	8.6	**842.70**	443.5	46.51	6.0	49.77	9.9	48.51	2.7	50.88	6.9	46.05	2.2	47.37	0.7
ILMN_1718033	LYPD5	44.27	0.1	61.20	16.9	106.35	13.0	**1951.04**	1216.2	55.10	3.4	89.77	13.3	52.32	5.2	66.97	15.7	56.74	7.3	70.75	3.8
ILMN_2388484	MAP2	59.78	0.3	82.31	24.2	98.60	18.6	**1171.85**	722.7	58.57	6.7	79.79	3.2	47.16	1.8	53.15	3.2	113.04	27.6	107.14	8.1
ILMN_1801610	METRNL	536.69	0.3	591.33	251.6	63.46	4.9	**694.96**	345.5	62.40	7.5	74.02	12.3	117.07	24.8	138.34	14.0	131.82	14.2	115.86	13.4
ILMN_2371911	MUC1	143.36	0.6	202.42	53.2	74.92	21.0	**1000.48**	263.6	1568.28	1073.0	2580.89	1351.5	73.54	7.5	77.76	8.5	49.12	2.2	60.31	16.0
ILMN_1662358	MX1	718.26	1.9	**10730.74**	7371.9	5578.05	4398.0	17909.55	2646.0	2081.13	1510.0	13433.93	4637.0	20289.26	6972.0	19984.79	6859.6	309.04	70.4	2534.77	893.0
ILMN_1713397	NCCRP1	50.95	0.2	53.63	2.8	105.54	46.3	**2133.37**	612.5	100.53	41.2	61.27	4.9	447.73	398.4	163.58	124.6	44.15	0.6	46.92	0.4
ILMN_1674063	OAS2	253.92	0.6	**4329.48**	1836.7	3083.40	1274.9	7362.87	2188.3	117.20	106.3	**1897.39**	368.1	3043.40	1688.0	3615.51	1555.6	202.56	89.1	1208.07	194.9
ILMN_1693192	PI3	48.98	0.1	44.36	1.9	965.35	557.7	**11419.86**	2677.6	283.62	195.0	65.43	10.3	50.67	3.8	48.72	2.4	43.94	2.9	42.29	0.7
ILMN_1815023	PIM1	1176.16	1.1	901.48	359.0	354.74	45.6	**4939.44**	2578.8	747.24	343.8	943.70	416.6	102.64	21.6	96.69	19.9	87.08	32.5	96.57	23.4
ILMN_2376108	PSMB9	187.57	0.7	**2542.10**	516.9	243.36	9.9	1042.49	419.2	288.94	29.2	2723.48	655.0	403.61	274.2	1086.98	313.4	78.08	47.5	**1358.51**	1202.1
ILMN_1800091	RARRES1	52.02	0.2	118.93	82.1	48.70	2.1	**1661.48**	1005.2	89.68	18.2	193.27	89.9	55.11	6.2	58.95	2.3	62.65	6.4	69.09	10.6
ILMN_1701613	RARRES3	286.89	0.7	**12942.20**	5804.9	128.36	21.7	**8772.33**	2324.2	2348.77	337.8	**28950.28**	602.9	171.05	44.9	383.49	106.1	62.17	22.9	**1809.49**	1367.0
ILMN_1683179	RRAD	86.19	0.6	63.27	2.1	69.76	9.0	**1201.41**	880.7	1611.67	1019.4	1444.14	481.5	45.99	4.5	47.28	3.5	56.15	1.7	53.73	0.5
ILMN_1801216	S100P	51.84	0.3	104.19	117.8	58.75	11.5	**12726.25**	1851.0	85.61	34.5	72.25	29.3	49.15	4.8	43.18	1.8	42.09	2.0	41.94	1.5
ILMN_1712759	SBSN	244.46	1.0	182.80	167.6	300.96	100.3	**14248.09**	3514.4	51.16	3.9	47.55	3.8	53.46	2.9	51.97	3.7	42.26	0.9	44.32	2.9
ILMN_1740917	SCNN1B	52.96	0.1	55.99	6.6	62.13	5.3	**696.83**	247.9	48.70	3.7	55.12	5.0	54.35	1.6	52.32	2.7	50.34	3.9	51.08	1.6
ILMN_2114720	SLPI	51.63	0.2	50.64	4.5	597.01	197.4	**6506.51**	739.5	2139.08	1426.8	2184.73	951.3	49.57	2.3	44.48	1.3	42.82	3.4	43.37	0.4
ILMN_1716591	SPRR1A	38.85	0.2	42.92	9.3	266.78	200.3	**6084.44**	5091.9	54.15	20.9	44.65	8.6	48.47	6.0	45.33	11.2	97.59	11.8	93.50	15.3
ILMN_2191967	SPRR2D	44.58	0.2	44.37	2.3	420.01	416.6	**13954.08**	12217.5	44.87	3.5	46.25	4.0	44.24	4.0	44.30	1.6	57.09	1.7	52.51	3.3
ILMN_1810835	SPRR3	50.74	0.1	50.81	2.4	85.75	37.2	**5981.57**	7827.6	49.48	1.7	52.13	5.5	52.04	5.8	55.10	2.4	46.01	2.2	42.43	0.2
ILMN_1727589	SULT2B1	51.65	0.0	51.75	5.2	85.03	13.7	**1457.46**	235.2	53.49	7.5	52.54	3.6	69.51	16.2	68.01	8.1	44.97	3.0	48.06	4.8
ILMN_1751079	TAP1	1236.54	0.5	**13773.11**	3210.4	1972.73	192.8	**25648.96**	2364.5	1434.62	544.7	**15504.01**	1948.7	3833.53	1618.3	14098.87	1982.3	654.16	334.6	**10409.28**	5849.2
ILMN_1770922	TMEM45A	758.53	0.2	813.31	134.5	269.60	62.2	**2767.81**	917.2	73.13	27.7	53.04	3.3	139.19	49.0	122.61	33.8	137.64	90.3	126.11	71.7
ILMN_1758418	TNFSF13B	74.69	0.4	697.88	321.1	57.43	1.9	180.66	74.8	65.40	12.7	**819.94**	7.5	75.92	16.8	226.77	79.1	60.31	3.9	260.83	9.5
ILMN_1678841	UBD	49.53	0.1	68.58	16.1	50.81	13.0	**1960.63**	804.8	150.86	117.1	**5551.24**	826.5	47.89	2.2	100.32	33.1	73.61	16.0	**1967.35**	291.8
ILMN_1727271	WARS	1514.53	0.5	**20408.54**	7452.5	689.49	104.2	**30022.06**	501.3	913.46	79.7	**18692.93**	2211.8	6765.34	1591.4	27572.74	1562.6	914.36	61.8	**14680.06**	11560.4

Probe ID[a]	Gene[b]	FB[c]		FB + IFN-γ[d]		KC[e]		KC + IFN-γ[f]		PTEC[g]		PTEC + IFN-γ[h]		MEL[i]		MEL + IFN-γ[j]		HUVEC[k]		HUVEC + IFN-γ[l]	
		AVG[m]	SD[m]	AVG[n]	SD	AVG	SD	AVG	SD	AVG	SD	AVG	SD	AVG	SD	AVG	SD	AVG	SD	AVG	SD
ILMN_2337655	WARS	1370.47	0.3	13355.12	3647.9	708.45	93.3	**20061.52**	3727.7	996.92	110.2	**16604.77**	3358.1	8180.00	4406.7	22874.17	4982.5	1265.00	190.3	**15696.13**	8662.5
ILMN_2079042	WFDC5	47.16	0.1	46.81	1.6	144.72	22.5	**1549.36**	417.2	73.51	50.8	49.54	5.7	46.09	5.5	48.74	5.9	52.45	0.1	49.06	0.8
ILMN_1742618	XAF1	271.66	0.4	890.16	340.1	212.41	108.6	524.67	213.1	86.31	40.4	**1080.09**	306.0	666.42	19.6	1138.79	305.8	180.92	132.4	518.87	198.4

[a] Illumina probe IDs as included in the microarray database. [b] Official gene symbols are depicted for genes that are >10-fold up-regulated by IFN-γ in at least one non-hematopoietic cell type. [c] Fibroblasts (FB) cultured without IFN-γ. [d] Fibroblasts (FB) cultured with 100 IU/ml IFN-γ for 4 days. [e] Keratinocytes (KC) cultured without IFN-γ. [f] Keratinocytes (KC) cultured with 100 IU/ml IFN-γ for 4 days. [g] Proximal tubular epithelial cells (PTEC) cultured without IFN-γ. [h] Proximal tubular epithelial cells (PTEC) cultured with 100 IU/ml IFN-γ for 4 days. [i] Melanocytes (MEL) cultured without IFN-γ. [j] Melanocytes (MEL) cultured with 100 IU/ml IFN-γ for 4 days. [k] Human umbilical vein endothelial cells (HUVEC) cultured without IFN-γ. [l] Human umbilical vein endothelial cells (HUVEC) cultured with 100 IU/ml IFN-γ for 4 days. [m] AVG and SD are the average and standard deviation of probe fluorescence as measured within the sample group, respectively. [n] Values in grey and bold indicate that the average probe fluorescence as measured in the sample group after culturing with IFN-γ is >10-fold higher than the fluorescence measured in the same samples cultured in the absence of cytokines.
